# Biased random walk model for the prioritization of drug resistance associated proteins

**DOI:** 10.1038/srep10857

**Published:** 2015-06-03

**Authors:** Hao Guo, Jiaqiang Dong, Sijun Hu, Xiqiang Cai, Guangbo Tang, Jianhua Dou, Miaomiao Tian, Fuchu He, Yongzhan Nie, Daiming Fan

**Affiliations:** 1 State Key Laboratory of Cancer Biology and Xijing Hospital of Digestive Diseases, Fourth Military Medical University, Xi’an, P. R. China; 2 State Key Laboratory of Proteomics, Beijing Proteome Research Centre, Beijing Institute of radiation Medicine, Beijing, P. R. China

## Abstract

Multi-drug resistance is the main cause of treatment failure in cancer patients. How to identify molecules underlying drug resistance from multi-omics data remains a great challenge. Here, we introduce a data biased strategy, ProteinRank, to prioritize drug-resistance associated proteins in cancer cells. First, we identified differentially expressed proteins in Adriamycin and Vincristine resistant gastric cancer cells compared to their parental cells using iTRAQ combined with LC-MS/MS experiments, and then mapped them to human protein-protein interaction network; second, we applied ProteinRank to analyze the whole network and rank proteins similar to known drug resistance related proteins. Cross validations demonstrated a better performance of ProteinRank compared to the method without usage of MS data. Further validations confirmed the altered expressions or activities of several top ranked proteins. Functional study showed PIM3 or CAV1 silencing was sufficient to reverse the drug resistance phenotype. These results indicated ProteinRank could prioritize key proteins related to drug resistance in gastric cancer and provided important clues for cancer research.

Multi-drug resistance (MDR) is the main cause of the failure of anticancer chemotherapies and has been studied for decades. Wet-lab experiments, including high throughput genomic and proteomic quantitative analysis, have established a large body of knowledge regarding MDR in cancer cells during chemotherapy, and we now understand that one or a combination of the following mechanisms contribute to MDR development^1^,^2^,^3^: (a) increased drug efflux and/or decreased drug uptake usually facilitated by drug transporters, such as members of the well-known ATP-binding cassette (ABC) family, ABCB1 (P-glycoprotein, also known as P-gp or MDR1) and ABCC1 (also known as MRP1); (b) increased drug detoxification by metabolizing toxic drugs into low- or non-toxic agents by the CYP450 enzymes or the glutathione S-transferase; (c) altered drug-target expression that is exemplified by a mutation or amplification of the binding sites for certain chemotherapeutics; and (d) resistance to apoptosis. Our lab has previously established two chemo drug resistant variant gastric cancer cell lines SGC7901/ADR and SGC7901/VCR by stepwise induction and identified a serial of molecules involved in the drug resistance in gastric cancer cells (GCCs). For example, an increased expression of ZNRD1 was found in both Adriamycin (ADR) and Vincristine (VCR) resistant GCCs and its inhibition could dampen the expression of P-gp and sensitize cells to chemo drugs^4^. Instead, suppression of GAS1 could result in epirubicin resistance in GCCs^5^. To explore the potential biomarkers of MDR in gastric cancer, we screened the differentially expressed cell membrane glycoproteins in drug resistant cell lines and found an enhanced N-glycosylation of P-gp protein^6^. Moreover, we also found that miR-15 b and miR-16 were able to control the cell apoptosis in GCCs^7^ by targeting BCL2 and miR-508-5 p was sufficient to reverse the chemo resistance phenotype in GCC^8^ by direct targeting ABCB1 and ZNRD1. However, these are far beyond the understanding of biological processes engaged in the development of MDR in cancer cells. The interplay between the MDR related molecules and the core regulatory network that controls the MDR phenotype still remain great challenges for the cancer research.

Recently, the emergence of large-scale interactome datasets has encouraged network-based systematic strategies that take advantage of multiple ‘-omics’ data generated across cell lines and tissues. These methods were designed to uncover the molecular interacting mechanism of drugs and drug targets^9^,^10^, to discover multi-target intervention drugs^11^, to prioritize disease related genes^12^,^13^, to identify dysregulated pathways in cancer cells^14^ and to predict various cancer outcomes^15^,^16^. Among these strategies, random walk (RW) algorithms covering the complex biological network is one of the most effective methods to infer phenotype associated genes or proteins. A RW model is actually a simplified variant of the PageRank algorithm used by Google’s search engine^17^. By walking on the protein-protein interaction (PPI) network or other biological networks, RW identifies proteins not only directly connected with known disease genes but also topologically similar with known disease genes. Using PPI networks and prior information of a disease, a RW and its modified versions have been shown to perform better than other methods in the identification of disease related proteins and subnetworks^18^,^19^,^20^. Based on this RW algorithm, Erten *et al.* developed a new method, named VAVIEN, to prioritize candidate disease genes by comparing their topological similarity profiles generated by a RW with known disease genes in the PPI network^21^. The results indicated that VAVIEN outperformed several popular methods, including a RW model utilizing only PPI information.

In this study, we present a novel proteomic-data biased random-walk strategy named ProteinRank to prioritize drug-resistance related proteins. The following important features differentiate this method from previously described algorithms: (i) both quantitative proteomics data and PPI information are used; (ii) both known and unknown proteins related to drug resistance in cancer cells are ranked; and (iii) valuable insights of proteins potentially associated with drug resistance are recommended. We tested ProteinRank on quantitative proteomic datasets obtained from ADR resistant gastric cancer cell line (SGC7901/ADR), VCR resistant gastric cancer cell line (SGC7901/VCR) and their parental SGC7901 cell line. Through leave-one-out cross-validation, ProteinRank displayed a greater area under the ROC curve and a higher average rank of reported drug resistance related proteins compared to the VAVIEN strategy either in ADR or VCR study. According to the rankings generated by ProteinRank, we focused on several highly ranked proteins which might be involved in ADR resistance in GCCs. Further validation with qRT-PCRs and western blots confirmed that several top ranked proteins recommended by ProteinRank displayed altered mRNA and protein expression levels, such as HAX1, PIM3 and CAV1. RNAi based functional validation indicated that PIM3 and CAV1 are involved in the acquisition and maintenance of the ADR resistant phenotype in GCCs.

## Results

### Retrieval of reported drug resistance associated proteins in gastric cancer

As there is no database that contains all the information about reported drug-resistance related proteins, two strategies were adopted to retrieve drug resistance related proteins in gastric cancer. Taking ADR study for example, thirteen non-redundant proteins linked to ADR (Accession Number: DB00997) were firstly collected from the DrugBank^22^ database which combines detailed drug data with comprehensive drug target, enzyme and transporter information. DrugBank is a unique cheminformatics resource that has been used by various computational models to derive drug or drug resistance related proteins^23^. This protein list contained 1 ADR targeted gene, 4 enzymes and 8 transporters. Second, GLAD4U^24^, a PubMed literature mining tool, was adopted to select ADR resistance related proteins in GCCs. As a result, 154 publications were examined and 6 proteins were ranked above the threshold and reported by at least 2 literatures. After combing the two list above and removing proteins with no interacting partners in the HPRD database, we finally obtained a set of 11 proteins that were considered by ProteinRank as known proteins related to ADR resistance (input seeds) in gastric cancer (Supplementary Table 1). Following the same procedure, we also retrieved 12 seed proteins that were associated with VCR resistance (Supplementary Table 2).

### Proteome profiling of drug resistant gastric cancer cell lines and construction of PPI network

To identify differentially expressed proteins (DEPs) in drug resistant cells, iTRAQ labeling was combined with an LC-MS/MS experiment to examine SGC7901/ADR, SGC7901/VCR and SGC7901 cell lines. As a result, 1183 proteins were identified with ≥95% confidence (Unused Score ≥ 1.3) and at least two peptides (Peptides 95% ≥ 2). Among these proteins, the expression levels of 130 and 165 proteins were significantly altered in SGC7901/ADR cells and SGC7901/VCR cells (P < 0.05; Supplementary Table 3 and Table 4) compared to SGC7901. Then, 39,204 binary interactions between 9,673 proteins were downloaded from the HPRD database (Release 9)^25^.

### Principles of the prioritizing strategy used by ProteinRank

**Figure 1:**
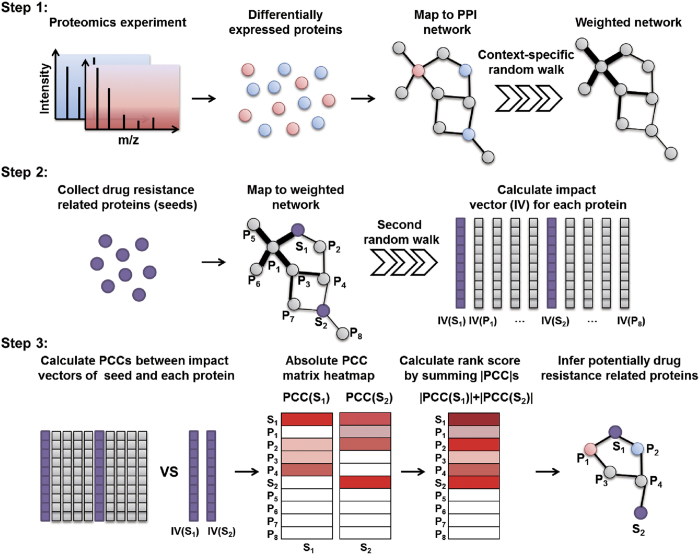
The general procedure of ProteinRank. A weighted network was generated by a random walk algorithm on a context-specific PPI network with fold change values of differentially expressed proteins (red, up-regulated; blue, down-regulated). Known drug resistance related proteins were collected from DrugBsank and the literature. All of the proteins in the weighted PPI network were regarded as candidate proteins. Their impact vectors were compared with seed impact vectors using PCC. By summing the absolute value of the PCCs of candidate proteins and seed pairs, ProteinRank can rank all candidate proteins. The top ranked proteins have similar impact patterns to seed proteins.

A recent study showed that proteins involved in identical diseases or biological pathways often share similar functions, and proteins with similar functions often interact^26^. Based on the observation that the expression level and position of each protein in the PPI network often affect their immediate interacting partner or even the entire pathway^12^, we developed ProteinRank to infer drug-resistance related proteins. The prioritization process in this strategy consists of three steps (Fig. 1). First, PageRank is able to provide a centrality measure that can reflect the topological importance or impact of the nodes in a complex network^27^. Therefore, we adopted a random walk with DEPs as input seeds to infer the impact abilities of each protein on the entire PPI network. Fold changes of these DEPs could propagate through the entire network by an iterative random walk. We then used these impact abilities to modify the PPI network and re-construct an edge-weighted, context-specific PPI network. This weighted network reflected the influence of the DEPs in the drug resistant cells. Second, based on the weighted network, we utilized known drug-resistance related proteins collected from DrugBank and literatures as seeds and implemented random walk again to derive the impact vector (IV) of each protein in the weighted network. An IV reflects the impact pattern of a protein in the network. Proteins with similar impact patterns in the network might be involved in similar biological processes. Finally, we compared the impact similarities between seed and other proteins in the network by calculating a Pearson correlation coefficient (PCC) between each pair of impact vectors. By summing the absolute PCCs, the total impact similarity (rank score) between candidate and the known seeds was obtained. A protein rank list was then generated according to the rank scores. A higher ranking indicated a more similar impact pattern to the known drug-resistance related proteins. A statistical P value for each rank score was then calculated by permutation test. Top ranked proteins were then considered as most concern candidates for further experimental verification.

### Leave-one-out cross-validation

To assess the performance of ProteinRank model in prioritizing proteins associated with drug resistance, we adopted a large scale leave-one-out (LOO) cross-validation^28^. In each validation run, a known seed protein was removed from the seed set and added to 99 control proteins which were randomly selected from the PPI network. These 100 proteins formed a test protein list. Then we used the proposed model to calculate the relevance of each protein in the list to the remaining seed set and generated a rank score for each protein. According to this score, the rank of each protein in the list could be determined. After each run, we obtained a ranking of the removed seed protein. The rank ratio was calculated as the division of the seed ranking over the size of test protein list, namely 100. For a given rank ratio threshold, the sensitivity was defined as the number fraction of seed proteins ranked above the particular threshold, whereas specificity was defined as the fraction of control genes ranked below this threshold. By varying the threshold from 0 to 1, we could plot receiver operating characteristic (ROC) curves. The area under the ROC curve (AUC) and the mean rank ratio (MRR) were calculated to measure the performance of the method. A larger AUC and a lower MRR indicate a better performance.

### Prioritizing performance evaluation based on ADR and VCR resistance study

Considering that the VAVIEN algorithm, a model which uses only PPI information and is similar to our strategy, outperformed the other random-walk based algorithms in ranking candidate disease genes, we adopted LOO cross-validation to compare the performance of VAVIEN with ProteinRank strategy biased to MS data from ADR and VCR resistance study. During each run of the cross-validation experiments, identical seed set and random control protein list were used by both two methods. All analyses were implemented on the same HPRD PPI network.

**Figure 2:**
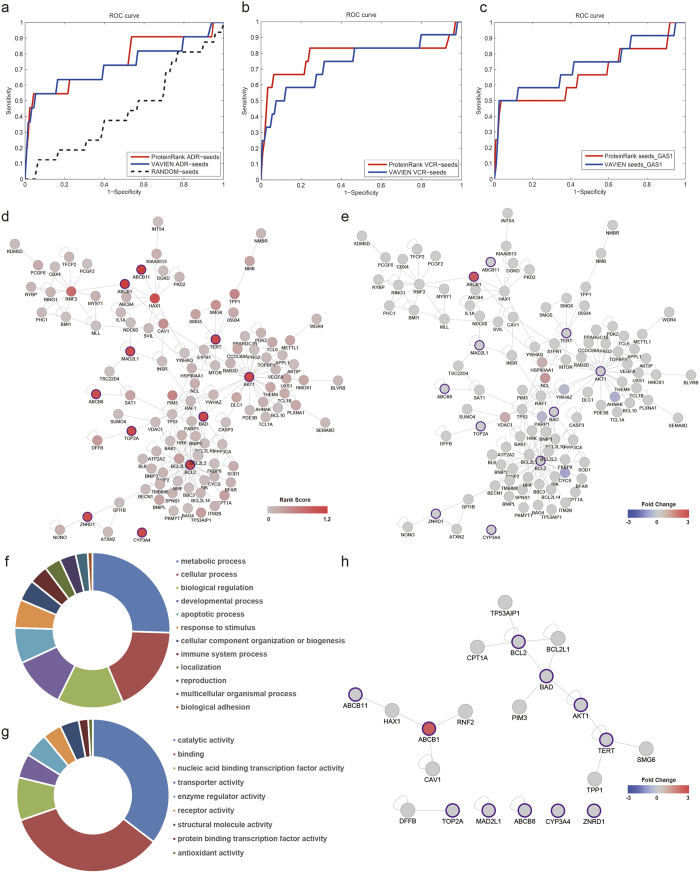
Performance evaluation and application of ProteinRank in drug resistance study. (**a**) ROC curve for ProteinRank and VAVIEN algorithm using 11 known ADR resistance related seeds obtained by LOO cross-validation. The dash curve represents the ROC of ProteinRank using random selected seeds from the whole network. (**b**) ROC curve for ProteinRank and VAVIEN algorithm using 12 VCR seeds. (**c**) ROC curve for ProteinRank and VAVIEN algorithm using 11 ADR seeds along with GAS1 gene. (**d**) Rank scores and interactions among the top 100 proteins ranked by ProteinRank using 11 ADR seeds and corresponding ADR MS data. The redder the node is, the more relevant the protein is to ADR resistance. Node with purple border is ADR seed. (**e**) Expression fold changes (Log2 transformed) of the top 100 proteins according to the proteomics data. ProteinRank recommended the most relevant proteins with ADR resistant seeds proteins, including both differentially and non-differentially expressed proteins. Node color represents the protein expression fold change identified by MS. Blue indicates reduced expression; Grey indicates no change; and red indicates over expressed. (**f**–**g**) Functional analysis of the top 100 proteins ranked by ProteinRank using PANTHER classification tool. The categorizations were based on the (**f**) Biological process and (**g**) Molecular function provided by PANTHER. (**h**) Interactions between the top 10 proteins ranked by ProteinRank and the 11 known ADR seeds.

For ADR study, based on the fold changes of 130 DEPs identified by MS and 11 known seeds, ProteinRank obtained an AUC of 0.7530 and an MRR of 0.2545 (Fig. 2a), while the AUC and MRR of VAVIEN are 0.7314 and 0.2764. We also assembled a randomly selected proteins list from the PPI network as the seed set. Cross-validation results showed that the AUC value for ProteinRank with random seeds was 0.4436 and the MRR was 0.5519. We performed another comparison between ProteinRank and VAVIEN using 165 DEPs identified from a VCR study and 12 known VCR resistance related proteins. LOO cross-validation results showed that ProteinRank had a better AUC (0.7883) and MRR (0.2200) than those of VAVIEN (0.7424 and 0.2650). The ROC curve was shown in Fig. 2b. These results demonstrated that ProteinRank had better performances in the prioritization of either ADR or VCR resistance related proteins than those of VAVIEN.

As our model is MS data biased, if one seed is not related or uncertain to ADR resistance, the LOO cross-validation might not tend to give a higher rank of this seeds. However, VAVIEN only utilize PPI information to rank candidates which are topologically similar to the seeds. As long as the candidate protein is connected (directly or indirectly) to the seeds, this protein might be highly ranked by VAVIEN. We have tested the performance of ProteinRank and VAVIEN using 11 ADR seeds along with GAS1 gene which was reported to be linked with epirubicin resistance (false seed). Results (Fig. 2c) showed that ProteinRank had a poor performance when taking GAS1 into account (AUC = 0.6700 and MRR = 0.33367), while the performance of VAVIEN was only slightly affected by the false seed (AUC 0.7256, MRR 0.2817). These results indicated that the MS data biased ProteinRank model tended to prioritize proteins associated with both known seeds and proteins with altered expression levels in the drug resistant cells, while VAVIEN only found proteins topologically similar to the seeds in the PPI network.

We compared the top 100 proteins ranked by ProteinRank (Supplementary Table 5) with VAVIEN (Supplementary Table 6) from ADR study. The results showed that 61 proteins were ranked by both methods. Among these ranked proteins, ProteinRank captured 7 differentially expressed proteins (NCL, HSP90AA1, AHNAK, YWHAZ, CYCS, PARP1 and VDAC1) identified in the MS experiment; whereas only one (AHNAK) was identified by VAVIEN. The rank order of AHNAK in the ProteinRank result was 29 and that in VAVIEN was 44. We also compared the top 100 candidates of VCR resistance related proteins for two methods (Supplementary Table 7 and Table 8). Among these candidates, ProteinRank found 11 proteins with altered expression level, such as ANXA11, CANX, NONO, IMPDH2, AHNAK, YWHAG, RTN4, PRKDC, PDCD6, ALDOA and FLNA, while VAVIEN only identified 5 DEPs (NONO, IMPDH2, AHNAK, ANXA11 and CANX). The DEPs identified by VAVIEN were all included in the top 100 protein list of ProteinRank, and the rankings of these proteins were much higher in ProteinRank. These results suggested the effectiveness of relying on proteomics data to uncover drug resistant proteins by using ProteinRank model.

### Prioritization of proteins associated with ADR resistance in gastric cancer using ProteinRank

Due to the limitation of MS experiments, among the known ADR resistance related proteins, only P-gp (encoded by the ABCB1 gene) was identified to be differentially expressed in the iTRAQ MS data. Moreover, most of the seeds and DEPs have little direct physical connections with each other (Supplementary Figure 1). It remains a challenge to infer the underlying relationship among known seed proteins and DEPs. Based on the expression fold changes and PPI network, ProteinRank discovers proteins with impact patterns similar to seed proteins in term of PCCs. By calculating the sum of absolute PCCs as rank scores, ProteinRank was able to recommend proteins that are potentially related to ADR resistance. The interaction map and rank scores for the 11 ADR seeds and top 100 proteins ranked by ProteinRank were shown in Fig. 2d. The expression fold change identified by MS for each protein was shown in Fig. 2e. By sorting the rank score of the proteins in this network, ProteinRank actually ranked both known and unknown proteins simultaneously. The rank scores for the top 100 proteins and 11 seeds were detailed in Supplementary Table 5.

To classify the function of these proteins, we adopted the PANTHER classification system^29^, which categorizes proteins into functional families and subfamilies with shared functions. PANTHER allows a more detailed and accurate association with the ontology terms of biological process, molecular function and biological pathways. The functional classification results of the top 100 unknown proteins ranked by ProteinRank demonstrated a high enrichment of 13 biological process terms (Fig. 2f) such as metabolic process, cellular process, biological regulation and apoptotic process, and 9 molecular function terms (Fig. 2g), including catalytic activity, binding, transcription regulator activity, etc., which were highly similar to the classification results of the proteins identified by MS experiment (Supplementary Figure 2). These results indicated that proteins recommended by ProteinRank were more likely to be functionally related to proteins with significant expression alterations.

ProteinRank prioritized both known seeds and unknown proteins that were related to ADR resistance in GCCs. The top 5 ADR resistant relevant seeds ranked by ProteinRank were ABCB1, ABCB11, AKT1, BAD and BCL2. These proteins are the most common molecules reported to be involved in drug-resistance associated cell apoptosis and drug transport processes^30^. As biologists always focus on most relevant molecules for further validation and functional exploration, the selecting range of top rank candidates are flexible, for example top 10 or top 100. We also provided a permutation P value for the rank score of each protein. The ranking orders and scores of the top 10 proteins along with the 11 seeds are listed in Table 1. An interacting map (Fig. 2h) of these proteins showed that HAX1 interacted with both ABCB1 and ABCB11 (also bind to ABCB4); RNF2 interacted with ABCB1; and PIM3 and TPP1 were both connected with apoptosis associated proteins, such as BAD, BCL2, AKT and TERT. These top ranked proteins have many connections with known seeds and hold important positions in the network. Several top ranking proteins were reported previously to be associated with drug resistance in cancer. For example, HAX1, RNF2, and CAV1 interact with ABCB1, and these proteins are also prioritized in the top 5 candidate list in VCR study using ProteinRank (Supplementary Table 7) and involved in chemoresistance in various carcinoma cells^31^. High expression levels of PIM3 can also mediate drug resistance to rapamycin in hematologic malignancies^32^.

### Validation of the ProteinRank recommended proteins in multi-drug resistant gastric cancer cell lines

**Figure 3:**
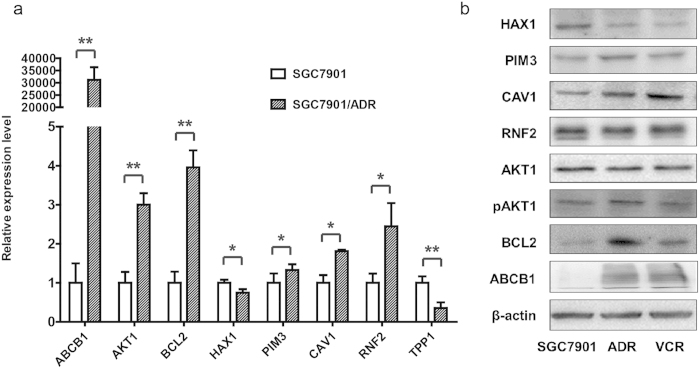
(**a**) Quantitative real-time PCR analysis of the top ranking proteins identified by ProteinRank. The fold changes shown are the expression levels of each gene in the SGC7901/ADR cell line relative to the SGC7901 cell line. Data are shown as the mean ± standard deviation. *P < 0.05, **P < 0.01. (**b**) Western blot verification of the top ranking proteins in the SGC7901, SGC7901/ADR and SGC7901/VCR cell lines.

To test whether the proteins recommended by ProteinRank were related to drug resistance in gastric cancer, we first verified the expression patterns of the top 5 ranked proteins obtained by ProteinRank. The qRT-PCR experiments were designed to detect the expression levels of the top 5 ranking HAX1, RNF2, PIM3, TPP1 and CAV1 genes in addition to the seed proteins ABCB1, AKT1 and BCL2 in both the SGC7901 gastric cancer cell line and the Adriamycin resistant SGC7901/ADR cell line (Fig. 3a). Significant expression alterations were displayed in all 8 genes: 6 genes were up-regulated (ABCB1, AKT1, BCL2, PIM3, CAV1 and RNF2) and 2 genes were down-regulated (HAX1 and TPP1).

The expression of these candidates at the protein level was further validated using western blot in both the VCR resistant SGC7901/VCR gastric cancer cell line and the SGC7901/ADR cell line (Fig. 3b). Higher expressions of BCL2, PIM3, CAV1 and increased phosphorylation of AKT1 (the protein expression level was not notably changed) were observed in the two drug resistant cell lines. A remarkable decrease in the expression of HAX1 was detected in SGC7901/ADR and SGC7901/VCR cells compared with SGC7901. No detectable RNF2 changes were observed. These verification results indicated that the top ranking proteins recommended by ProteinRank might maintain important roles in MDR mechanisms in gastric cancer and require further functional experiments.

**Figure 4:**
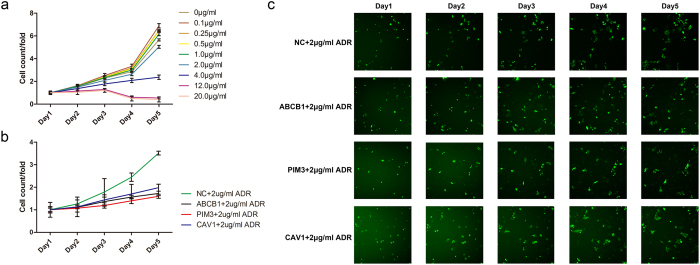
Functional validation of ProteinRank prioritized proteins. (**a**) SGC7901/ADR cells stably infected with the negative control virus were exposed to gradients of diluted Adriamycin and cell growth rates under different drug concentrations were measured. (**b**) The growth curve of SGC7901/ADR cells with 2 μg/ml Adriamycin when PIM3 and CAV1 were silenced. ABCB1 was selected as the positive control. (**c**) Representative images of the cellomics detection results.

To test whether ProteinRank recommended proteins function in the maintenance of the drug resistance phenotype of SGC7901/ADR cells, we evaluated the loss-of-function effects of two potential candidates, CAV1 and PIM3, on the drug induced growth inhibition of SGC7901/ADR cells. GFP-labeled shRNAs targeting CAV1, PIM3, ABCB1 (positive control, PC) and a negative control (NC) were stably infected, and the cell growth rates were measured by the ArrayScan VTI HCS Reader based on fluorescence intensity. The optimal drug concentration that distinguished the drug toxicity and loss-of-function gene effect were first explored with a gradient of diluted Adriamycin. We found that 2 μg/ml was the maximum concentration that does not affect the growth rate of SGC7901/ADR cells infected with the NC virus (Fig. 4a), and this concentration was selected for further measurement. We observed that the growth rates of cells infected with the ABCB1, CAV1 and PIM3 shRNAs were significantly retarded under 2 μg/ml Adriamycin compared with cells infected with the NC virus. The highest inhibitory effect was observed when PIM3 was silenced (Figs 4b,c).

## Discussion

Based on the notion that proteins involved in identical diseases or pathways might form highly interlinked sub-networks within the interactome network^12^,^33^, multiple network-based analyses have been proposed to uncover molecules and sub-networks associated with certain phenotype or specific condition. For example, by defining the topological closeness, effective computational models can successfully infer potential interactions of disease-gene^34^, drug-target^35^ or co-modules^36^ of drug-disease-gene based on the PPI network. Among these network analyses, RW algorithm has been proved to be effective either in disease gene^19^ or drug target discovery^23^. Here we presented a variant RW based model, named ProteinRank, to prioritize proteins associated with drug resistance. A key feature of this strategy is that ProteinRank could take advantage of multiple information sources from drug resistance study of cancer including measured expression fold changes of proteins, prior knowledge of well-studied known drug resistance related proteins and interaction networks among proteins. The goal of first random walk is actually to generate a context-specific network from proteomic data. Based on this weighted network, the second random walk could determine the impact pattern of each node in the network in terms of impact vector. By comparing the similarities of IVs between known drug resistance related proteins and proteins in the network, one can finally infer which protein has a similar impact pattern with known proteins under the context of drug resistant phenotype.

We validated this method in a large scale leave-one-out cross-validation study of ADR and VCR resistance in gastric cancer. Compared to topological similarity based method, VAVIEN, validations using identical seeds and identical randomly selected control-protein sets suggested that ProteinRank outperformed VAVIEN. This outperformance was noted in terms of both the area under the ROC curve and the mean ranking ratio of known ADR resistance related protein. The functional classification results suggested that proteins recommended by ProteinRank and identified by iTRAQ MS were involved in similar biological processes and were functionally related. These results indicated that ProteinRank had a better performance in inferring drug resistance related proteins than topological analysis based model by integration of MS data and PPI information and could recommend proteins both functionally associated with significantly expressed proteins and topologically similar to known drug-resistance related proteins.

The study of ADR resistant gastric cancer cell lines demonstrated that ProteinRank was capable of providing valuable insights into ADR resistance related genes. The qRT-PCR experiments detected significant changes in the mRNA expression levels of the 8 top ranking genes (ABCB1, AKT1, BCL2, HAX1, PIM3, TPP1, CAV1 and RNF2) in ADR resistant gastric cells compared to parental cell lines. Western blotting further verified the altered protein expression levels of HAX1, PIM3 and CAV1 in both ADR and VCR resistant cells. This finding suggests a valuable insight into MDR mechanisms in gastric cancer. Notably, we found the active, phosphorylated AKT1 was elevated in the western blot analysis; however, total AKT1 remained stable in the drug resistant variants. This partially explains the lack of detectable changes in AKT1 observed in the iTRAQ MS/experiment. Post-translational modification maintains an important role in determining protein activity. However, describing these modifications usually requires specific methods, for example, a commonly used antibody based strategy. The specificity of the methods limits the value of post-translational modifications in routine quantitative proteomics analysis. To an extent, ProteinRank can bridge this technical gap. Overall, verification results demonstrated that proteins obtained by ProteinRank could suggest valuable insights into and guide the further functional study of ADR resistance mechanisms in gastric cancer.

To confirm that ProteinRank prioritized proteins actually influence Adriamycin resistance in GCCs, we selected the overexpressed PIM3 and CAV1 for functional experiments. The results showed that silencing either of these two genes was sufficient to partially reverse the Adriamycin-resistant phenotype of SGC7901/ADR cells. Therefore, ProteinRank successfully recommended functional proteins involved in the maintenance of Adriamycin resistance in GCCs.

Since the discovery of the first MDR related molecule, the P-gp protein, a large number of investigations have broadened our insight into the mechanisms involved in the development of drug resistance. In addition to the family of ABC transporters which are regarded as a multi-drug efflux pump, several metabolic enzymes that detoxified cytotoxic agents before they reached their targets and regulators or executors of apoptosis have also been considered as classical MDR molecules. In the present study, we observed altered mRNA and protein expression levels of HAX1, PIM3 and CAV1 in drug-resistant GCCs and deduced that these proteins might fall into this category of drug-resistance related molecules.

HAX1 is a 34-kDa protein and has been widely reported to be involved in the regulation of apoptosis, mRNA processing, cell motility and calcium homeostasis^37^,^38^,^39^. Generally, HAX1 was considered to be an anti-apoptotic protein. HAX1 has been reported to inhibit the activity of caspase 3 and enhance the stability of the anti-apoptotic XIAP against proteosomal degradation. Han *et al.*^40^ showed that over-expression of HAX1 inhibited caspase 9 and protected cardiac myocytes from apoptosis. Moreover, Sun *et al.*^31^ reported that HAX1 promoted cisplatin chemoresistance in esophageal squamous carcinoma cells. However, in our present study, decreased HAX1 expression was observed. Therefore, the chemoresistance-promoting role of HAX1 might be attributed to other functions. Previously, Ortiz *et al.*^41^ identified and validated HAX1 as a direct binding partner for several ABC family members, including MDR1 (ABCB1, P-gp), MDR2 (ABCB4), and BSEP/SPGP (ABCB11). They found that HAX1 was co-localized with BSEP and MDR1 in the apical membrane and involved in the internalization of these transporters from the membrane to the cytoplasm. The down-regulation of HAX1 increased the BSEP levels in the apical membrane. Therefore, reduced HAX1 expression in the drug-resistant GCCs may also contribute to the transportation and internalization of the drug transporters and affect the efflux of the drug from the cytoplasm. Further investigations are needed to uncover the multiple functions of HAX1 in the degradation of transporters and multi-drug resistance.

PIM3 has been reported to participate in the development of various cancers, including hematological malignancies, gastrointestinal tumors, pancreatic cancer and hepatocellular carcinoma. Forshell *et al.*^42^ reported that PIM3 was a direct target of oncogene c-Myc and played a role in supporting the viability of Myc-induced B-cell lymphomas. PIM3 mainly functions by phosphorylating downstream genes that are involved in cell cycle progression, protein translation and programmed cell death. One of the substrates of PIM3 is the tumor suppresser gene pro-apoptotic BCL2 family member BAD. PIM3 was reported to be capable of predominantly phosphorylating BAD on Ser112, leading to the release of the anti-apoptotic BCL-xL, preventing BAD mediated apoptosis^43^.

The role of CAV1 in drug resistance is controversial relying on the tumor types and chemotherapy drugs. CAV1 overexpression has been shown to be associated with drug resistance in non-small cell lung cancers and was reversed in oral squamous cell carcinoma. Shajahan *et al.*^44^ reported that the phosphorylation of a tyrosine in caveolin-1 (Tyr-14) increased the sensitivity of breast cancer cells to paclitaxel, and the mechanisms that were involved in this process included the inhibition of BCL2 and BCL-xL by a c-Jun N-terminal kinase (JNK).

Besides the advantages, there are still some limitations in our model. First of all, the model is limited to the quantity and quality of proteome and interactome data. The construction of the weighted network in the first step of ProteinRank depends on the numbers and expression fold changes of proteins identified by MS experiments and the completeness of protein interactions. The size of MS dataset and PPI network used in the model is still relatively small. Second, some prior knowledge of drug resistance related proteins is needed in our model. A well curated known seed proteins list will improve the accuracy of our model. Third, unreliable proteomic data or inaccurate prior knowledge will result in false positive candidates. Our future work will employ a new mass spectrometry (for example Q-Exactive) with higher resolution and sensitivity to generate a more comprehensive protein expression profile and utilize a larger curated PPI network by integrating multiple data source, such as BioGRID, MINT and STRING.

In conclusion, we developed a systematic strategy named ProteinRank to calculate the relevance of candidate proteins to known drug-resistance associated proteins based on proteomic data and PPI networks and demonstrated its application in the discovery of new targets to overcome drug resistance. The proposed approach sheds light on the study of the integration of multi-omics data and network analysis. It is also a promising and helpful tool to generate new biological hypotheses in the study of drug resistance in cancer.

## Methods

### The procedure of the ProteinRank algorithm

A random walk is a simplified version of PageRank, which is the core algorithm of Google’s search engine. Web pages that highly connect with notable web pages will be assigned a high score by PageRank. Similarly, ProteinRank adopted a random walk in a PPI network to infer which proteins have the largest impact on the entire network and to determine which proteins are relevant to drug resistance in cancer cells. We followed a general implementation of a random walk in an iterative form^19^:


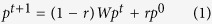


Where *W* represents the column-normalized network adjacency matrix, *p*^*t*^is the importance vector in which the *i*-th element represents the importance score for protein *i* at time step *t*, *r* is the restart probability which is set to 0.4 in this work (selection of r was detailed in the Supplementary Methods), and *p*^*0*^ is the initial importance vector.

To capture the importance with respect to a set of differentially expressed proteins between the case and control groups obtained by proteomics analysis, we biased the initial importance vector *p*^*0*^ to the expression fold changes (without Log2 transformed) of proteins in the first step of ProteinRank. This method was inspired by Topic-Sensitive PageRank^27^ and is equivalent to allowing the random walk algorithm to begin from each protein with different probabilities according to the protein expression fold changes. The impact value calculated by this context-specific random walk reflects the importance of each protein in the network. Let *G* = (*V, E*) be a PPI network, vector V denotes all proteins (nodes) in the network, and vector E denotes all interactions (undirected edge) among proteins. For a given protein, *v*_*i*_ ∈ *V*, *p*(*v*_*i*_) represents the impact value obtained by context-sensitive random walk based on the protein expression fold changes. We define the weight *w*_*ij*_ of the edge from node *i* to node *j* as (2):


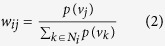


Where *N*_*i*_ is the node set of immediate interacting neighbors of node *i*. The greater the *w*_*ij*_, the more likely node *i* will interact with node *j*. The weighted PPI network can be represented in an adjacency matrix form (3):


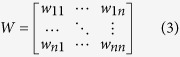


In the second step of ProteinRank, an impact vector (IV) which was also adopted in pathway analysis^20^ was calculated to determine the impact pattern of a given protein on the whole network. An IV of a given protein, *u*, which was denoted by *p*_*u*_, was calculated by rerunning the random walk process on the weighted network with a starting vector *p*_*u*_^*0*^ defined as (4):


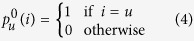


We calculated the impact vector for each protein in the network by using the random walk iteration equation described above. The IV derived from this step reflects the influence of a protein based on its impact and topological centrality.

In the third step of ProteinRank, we compared the impact profiles of two proteins, u and v, by calculating the Pearson correlation coefficient (PCC) between *p*_*u*_ and *p*_*v*_, denoted by Corr (*p*_*u*_, *p*_*v*_):


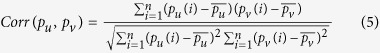


Where *p*_*u*_(*i*) is the *i*-th element of impact vector *p*_*u*_; 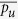 is the mean value of vector *p*_*u*_; and n is the total number of proteins in the network. This methodology is also used by VAVIEN to compare the topological similarity of two proteins^21^.

We then prioritize the proteins according to the sum of the absolute value of the PCC between the impact vector and those of known seeds. The rank score of protein *i* can be represented as (6):


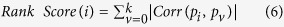


Where *p*_*i*_ is the impact vector of protein *i*; *p*_*v*_ is the impact vector of the seed protein *v*; and *k* is the size of seed set. A larger rank score indicated a stronger correlation with the impact patterns of the seed set. A statistical significance for each rank score was then calculated by permutation test (detailed in Supplementary Methods.).

The main procedure mentioned above was programmed in MATLAB code (which is available at http://cbskl.fmmu.edu.cn/ProteinRank/index.html.) and tested on a PC with a CPU of Inter(R) Core(TM)2 Duo E7400 @2.8 GHz and 4 GB of RAM.

### Cell culture, RNA isolation and qRT-PCR

The human gastric cancer cell line SGC7901 was obtained from the Academy of Military Medical Science, and its multi-drug resistant variants SGC7901/VCR and SGC7901/ADR were established and maintained in our laboratory. All cells were maintained in RPMI-1640 medium (Hyclone, Logan, Utah, USA) supplemented with 10% fetal bovine serum (Gibco, Carlsbad, CA, USA), 100 U/ml penicillin sodium and 100 μg/ml streptomycin sulfate at 37 °C in humidified air containing 5% CO_2_. To maintain the MDR phenotype, Vincristine and Adriamycin were added at final concentrations of 1 μg/ml and 0.5 μg/ml, respectively, to the culture media of SGC7901/VCR and SGC7901/ADR cells.

Total RNA was extracted from the cultured SGC7901 and SGC7901/ADR cells using TRIzol reagent (Invitrogen Life Technologies, Carlsbad, CA, USA) according to the manufacturer’s instructions. The concentration of total RNA was measured by SmartSpec Plus (BioRad, Hercules, CA, USA) at 260 nm. Five hundred nanograms of total RNA was reverse transcribed into cDNA with PrimeScript RT Master Mix Perfect Real Time (Takara, Dalian, Liaoning, China). qRT-PCR was performed in triplicate using the SYBR^®^ Premix Ex Taq™ (TliRNaseH Plus) kit (Takara, Dalian, Liaoning, China) at an annealing temperature of 55 °C. The relative amount of each mRNA that was monitored was normalized to beta-actin mRNA. The fold-change for each mRNA from SGC7901/ADR cells relative to the SGC7901 cells was calculated by using the 2^−ΔΔCt^ method. The primers used are listed in Table 2. A Student’s *t*-test was adopted to analyze the difference between the relative means of mRNA expression in the two cell lines.

### Cell lysis and protein quantitation

Cell protein was extracted as previously described. Briefly, 1 × 10^7^ cells were harvested and lysed in RIPA (Beyotime, Shanghai, China) containing protease inhibitors (Roche, Mannheim, Baden-Württemberg, Germany). The protein concentrations were determined using a BCA protein assay kit (Beyotime, Shanghai, China).

### iTRAQ labeling

The 8-plex iTRAQ reagents and buffers were obtained from AB SCIEX (Foster City, CA, USA). Protein samples (200 μg) from each cell line were treated with cold acetone for 4 hours at −20 °C. According to the manufacturer’s instructions, the precipitated pellets were then dissolved with a dissolution buffer, 6 M Urea and denaturant buffer. The samples were then reduced with a reducing reagent for 1 h at 37 °C; a cysteine-blocking reagent was then added, and the samples were incubated for 10 min at room temperature. A total of 10 μg of trypsin (Promega, Madison, WI, USA) was added to each of the samples and were digested overnight at 37 °C.

The volume of each was reduced to 30 μL using a SpeedVac (Christ, Osterode, Niedersachsen, Germany). iTRAQ reagents were dissolved in 70 μL of isopropanol and then mixed with each sample. After 3 h of incubation at room temperature, all the labeled samples were pooled together. The protein samples were labeled as follows: SGC7901 with 113, SGC7901/ADR with 114 and SGC7901/VCR with 115.

### LC-MS/MS Analysis

The iTRAQ-labeled mixture was first separated by an ICAT^TM^ strong cation-exchange cartridge (AB SCIEX, FosterCity, CA, USA) according to the manufacturer’s instructions. After being desalted by Ultramicrospin^TM^ columns (The Nest Group, Southborough, MA, USA), the eluted fractions were spotted and mixed with a MALDI matrix solution (5 mg/mL α-Cyano-4-hydroxycinnamic acid (CHCA) in 70% acetonitrile and 0.1% v/v TFA) with the nano-LC 1D plus (Eksigent, Redwood, CA, USA).

The sample spots were analyzed using a 5800 MALDI TOF/TOF Analyzer (AB SCIEX, FosterCity, CA, USA). Seven hundred and fifty laser shots were accumulated from each sample well, and the precursor ions were selected from MS spectra ranged from 800 to 4000 Da. MS/MS analysis was achieved with air used as the collision gas with a collision energy of 2 kV.

All MS data were analyzed using ProteinPilot software v4.0.8085 (AB SCIEX, FosterCity, CA, USA) using the Paragon algorithm 4.0.0.0 searched against the Uniprot human protein database.

### Western blot assay

Proteins were separated by sodium dodecyl sulfate–polyacrylamide gel electrophoresis (SDS–PAGE) and then transferred to a nitrocellulose filter membrane (NC, Pall Corporation, Pensacola, FL, USA). The membranes were blocked with 5% non-fatty milk and then probed with antibodies against RNF2 (1:500, Abcam, Cambridge, MA, USA), PIM3 (1:500, Abcam, Cambridge, MA, USA), HAX1 (1:500, Santa Cruz Biotechnology, Santa Cruz, CA, USA), AKT1 (1:1000, Cell Signaling Technology, Beverly, MA, USA), pAKT1 (1:1000, Cell Signaling Technology, Beverly, MA, USA), BCL2 (1:500, Immunoway, Newark, DE, USA) and β-actin (1:100000, Sigma, Louis, MO, USA). After washing with PBST (pH 7.4), the blots were incubated with horseradish peroxidase-conjugated secondary antibodies (1:2000, Zhongshan, Beijing, China). An ECL kit (Millipore, Billerica, MA, USA) was used to detect the intensity of each protein.

### shRNA-mix lentivirus production and SGC7901/ADR cell infection

The shRNA-mix lentiviruses were purchased from GeneChem and they were produced in the following method. Briefly, four pLKO.1 vectors containing short hairpin sequences targeting the human PIM3 and CAV1, separately; the packaging plasmid; and the envelop plasmid were co-transfected into 293T packaging cells. The supernatant was harvested and concentrated forty hours after transfection, and the virus titers were measured. The SGC7901/ADR cells were infected with the shRNA-mix lentivirus as previously described. The four targeted sequences for PIM3 and CAV1 are: 5’-GCCGTCGCTGGATCAGATT-3’,5’-TGCTTCTCTACGATATGGT-3’, 5’-AGGACCTCTTCGACTTTAT-3’, 5’-AGGCGGACAAGGAGAGCTT-3’ and 5’-CCCACTCTTTGAAGCTGTT-3’, 5’-ACCTTCACTGTGACGAAAT-3’ 5’-CGTGGTCAAGATTGACTTT-3’, 5’-TTTGTGATTCAATCTGTAA-3’, respectively.

### Cellomics data acquisition and data analysis

Image acquisition in the green channel was performed using an ArrayScan II HCS Reader from Cellomics. Two-thousand cells were seeded on a 96-well plate. The number of cells was counted by the software supplied with the ArrayScan VTI HCS Reader for five days, and the cell counts were normalized to the cell count on the first day. The growth curves were then plotted.

## Additional Information

**How to cite this article**: Guo, H. *et al.* Biased random walk model for the prioritization of drug resistance associated proteins. *Sci. Rep.* **5**, 10857; doi: 10.1038/srep10857 (2015).

## Supplementary Material

Supplementary Information

## Figures and Tables

**Table 1:** Ranking order and scores of seed proteins and the top 10 proteins inferred by ProteinRank in ADR study.

**Rank**	**Gene Symbol**	**HPRD ID**	**RankScore**	**P Value**
1	ABCB1*	01370	1.1842	0.0000
2	ABCB11*	04436	1.1712	0.0001
3	AKT1*	01261	1.0594	0.0002
4	BAD*	04409	1.0535	0.0004
5	BCL2*	01045	1.0444	0.0003
6	TERT*	01754	1.0342	0.0004
7	CYP3A4*	00484	1.0055	0.0005
8	TOP2A*	00536	1.0051	0.0006
9	ABCB8*	10400	1.0046	0.0006
10	ZNRD1*	09601	1.0045	0.0010
11	MAD2L1*	03274	1.0031	0.0012
12	HAX1	12075	0.9779	0.0012
13	RNF2	07028	0.7204	0.0012
14	PIM3	15137	0.5205	0.0012
15	TPP1	06415	0.4990	0.0015
16	CAV1	03028	0.4267	0.0017
17	SMG6	06502	0.4097	0.0020
18	DFFB	03532	0.3495	0.0017
19	BCL2L1	02497	0.3374	0.0015
20	TP53AIP1	10397	0.3307	0.0021
21	CPT1A	02755	0.3307	0.0020

^*^Seed proteins retrieved from DrugBank and GLAD4U.

**Table 2:** Primer sequences used in the qRT-PCR experiment.

**Primer**	**Sequence**
TPP1 forward	TGGAAAGACTCTCGGAGCTG
TPP1 reverse	TCCGTAGGTCCTCCCACATA
CAV1 forward	TCTTCCAACACGTAGCTGCC
CAV1 reverse	GCCGTCAAAACTGTGTGTCC
BCL2 forward	GTCATGTGTGTGGAGAGCGT
BCL2 reverse	GCCGTACAGTTCCACAAAGG
ABCB1 forward	GGGAGCTTAACACCCGACTTA
ABCB1 reverse	GCCAAAATCACAAGGGTTAGCTT
AKT1 forward	AGCGACGTGGCTATTGTGAAG
AKT1 reverse	GCCATCATTCTTGAGGAGGAAGT
HAX1 forward	ATAGTCACCAGCCCAGGATCT
HAX1 reverse	CCTGGGAAACCTGGGAATCAA
PIM3 forward	AAGGACGAAAATCTGCTTGTGG
PIM3 reverse	CGAAGTCGGTGTAGACCGTG
RNF2 forward	TGAGGCTCGCCATATTGTGC
RNF2 reverse	GGTTGAGTTCCGTTTGTCTGC
Beta-actin forward	CATGTACGTTGCTATCCAGGC
Beta-actin reverse	CTCCTTAATGTCACGCACGAT
