# Supplementary material for: Biased random walk model for the prioritization of drug resistance associated proteins
Source: Sci Rep. 2015 Jun 3;5:10857. doi: 10.1038/srep10857 (PMC4454201; doi:10.1038/srep10857)
Supplement: Supplementary Information [file srep10857-s1.doc]

Biased random walk model for the prioritization of drug resistance associated proteins

Hao Guo^1,2§^, Jiaqiang Dong^1§^, Sijun Hu^1§^, Xiqiang Cai^1^, GuangboTang^1^, Jianhua Dou^1^, Miaomiao Tian^1^, FuchuHe^2*^, Yongzhan Nie^1*^, Daiming Fan^1*^

**Supplementary Methods**

**Selection of restart probability in random walk model**

The restart parameter r controls the return probability of each random step to seed nodes. If r is close to 0, the algorithm will provide a more comprehensive view of the network structure around the seed nodes. If r is close to 1, a restricted view of the neighborhood of seed nodes will be obtained^1^. The selection of r value seems empirical in different research. In disease gene prioritization research, random walk with r=0.7 was used to infer gene phenotype relationships^2^. In biological pathway or network motif identification research^3^, r value was set to 0.01. In VAVIEN model^4^, by calculating the average rank of the target gene after LOO cross validation, the author claims that VAVIEN appears to be stable in the range [0.3-0.9] of r and provides a best performance when r is set to 0.5. We also investigated the effect of r selection on the performance of ProteinRank using LOO cross validation. The performance here is measured in terms of the mean rank ratio of the seed protein among 100 candidates (1 seed and 99 random proteins), a lower value indicating better performance. ProteinRank appears to be robust in the range [0.4, 0.6] and reaches a best performance at r=0.4. Therefore, we set r=0.4 in ProteinRank model.

**Permutation Test**

A statistical P value of the sum of the absolute PCC has been calculated in terms of permutation test. In short, 1) the original paired correlation score of a protein i in the network is RankScore(seeds,i); 2) a new score RankScore(seeds,i’) is redefined randomly where i’ is randomly drew from the permutation set of {1,…,n} (n is the total number of the proteins in the network); 3) a new RankScore is calculated from the randomized data; 4) by repeating step 2) and 3) 50000 times, the P value equals to the proportion of the RankScores generated in step 3) which are greater than the original RankScore obtained in step 1). The significance level is set to 0.05.

**References**

1. El Dayeh, M. & Hahsler, M. in Computational Intelligence in Bioinformatics and Computational Biology (CIBCB), *2012 IEEE Symposium on* 229-236 (IEEE, 2012).

2. Li, Y. & Patra, J.C. Genome-wide inferring gene-phenotype relationship by walking on the heterogeneous network. *Bioinformatics* **26**, 1219-24 (2010).

3. Komurov, K., White, M.A. & Ram, P.T. Use of data-biased random walks on graphs for the retrieval of context-specific networks from genomic data. *PLoS Comput Biol* **6** (2010).

4. Erten, S., Bebek, G. & Koyuturk, M. Vavien: an algorithm for prioritizing candidate disease genes based on topological similarity of proteins in interaction networks. *J Comput Biol* **18**, 1561-74 (2011).

**Supplementary Information**

Supplementary Table 1. ADR related proteins collected from DrugBank and GLAD4U. Supplementary Table 2. VCR related proteins collected from DrugBank and GLAD4U. Supplementary Table 3. Proteins with altered expression levels in ADR resistant cells identified by iTRAQ-MS/MS listing the accession number; protein name; gene symbol; unused and % coverage for each protein; peptides (95%); iTRAQ ratios for 114:113 (SGC7901/ADR: SGC7901) and P Value. Supplementary Table 4. Proteins with altered expression levels in VCR resistant cells identified by iTRAQ-MS/MS listing the accession number; protein name; gene symbol; unused and % coverage for each protein; peptides (95%); iTRAQ ratios for 115:113 (SGC7901/VCR: SGC7901) and P Value. Supplementary Table 5. ProteinRank scores for the 11 seed proteins and the top 100 ranked proteins in ADR study. Supplementary Table 6. The top 100 ranked proteins using VAVIEN with 11 seeds in ADR sutdy. Supplementary Table 7. ProteinRank scores for the 12 seed proteins and the top 100 ranked proteins in VCR study. Supplementary Table 8. The top 100 ranked proteins using VAVIEN with 12 seeds in VCR study.

Supplementary Figure1. Expression levels and interactions among seeds and differentially expressed proteins identified by MS in ADR study.

**Supplementary Tables：**

**Table 1.** ADR related proteins (with at least 1 PPI) collected from DrugBank and GLAD4U

| **Gene Symbol** | **HPRD ID** | **DrugBank^1^** | **GLAD4U PubMed ID^2^** |
| --- | --- | --- | --- |
| TOP2A | 536 | Target | - |
| CYP3A4 | 484 | Enzyme | - |
| ABCB1 | 1370 | Transporter | - |
| ABCB8 | 4436 | Transporter | - |
| ABCB11 | 10400 | Transporter | - |
| ZNRD1 | 9601 | - | [18058465], [12795835] |
| MAD2L1 | 3274 | - | [20440596], [16214181] |
| TERT | 1754 | - | [18396642], [12579337] |
| BCL2 | 1045 | - | [18478476], [12579337] |
| AKT1 | 1261 | - | [20514451], [19062713] |
| BAD | 4409 | - | [18449891], [16911523] |

^1^ Protein list was retrieved from DrugBank under the category of doxorubicin.

^2^ Proteins (at least reported by 2 literatures) retrieved from GLAD4U tools was using a search key word: gastric cancer doxorubicin resistance.

**Table 2.** VCR related proteins (with at least 1 PPI) collected from DrugBank and GLAD4U

| **Gene Symbol** | **HPRD ID** | **DrugBank^1^** | **GLAD4U PubMed ID^2^** |
| --- | --- | --- | --- |
| TUBB | 1852 | Target | [23178631], [20699370], [20556576],  [18389626], [18058465] |
| TUBA4A | 1851 | Target | - |
| CYP3A4 | 484 | Enzyme | - |
| ABCB1 | 1370 | Transporter | - |
| ABCC2 | 3065 | Transporter | - |
| ABCB11 | 4436 | Transporter | - |
| SLC22A3 | 5328 | Transporter | - |
| RALBP1 | 9013 | Transporter | - |
| ZNRD1 | 9601 | - | [18058465], [17492506], [14726695], [12795835] |
| PPP1R1B | 5097 | - | [18058465], [17492506] |
| SRI | 1680 | - | [21109982], [18423116] |
| AKT1 | 1261 | - | [23743572], [17310852] |

^1^ Protein list was retrieved from DrugBank under the category of vincristine.

^2^ Proteins (at least reported by 2 literatures) retrieved from GLAD4U tools was using a search key word: gastric cancer vincristine resistance.

**Table 3.** Differentially expressed proteins in SGC7901/ADR cells compared with SGC7901 cells identified by iTRAQ-MS/MS

| **Accession** | **Protein Name** | **Gene**  **Symbol** | **Unused** | **%Cov** | **Peptides**  **(95%)** | **114:113^a^** | **PValue** |
| --- | --- | --- | --- | --- | --- | --- | --- |
| P21333 | Filamin-A | FLNA | 161.52 | 49.45 | 105 | 0.82 | 0.0000 |
| P06733 | Alpha-enolase | ENO1 | 90.45 | 86.41 | 76 | 0.61 | 0.0000 |
| P04406 | Glyceraldehyde-3-ph | GAPDH | 58.58 | 71.34 | 53 | 0.68 | 0.0005 |
| P07900-2 | Isoform HSP90AA1-2 of Heat shock protein HSP 90-alpha | HSP90AA1 | 31.05 | 54.10 | 48 | 1.23 | 0.0086 |
| Q09666 | Neuroblastdifferentiation-associated protein AHNAK | AHNAK | 66.13 | 46.37 | 43 | 0.73 | 0.0000 |
| P08670 | Vimentin | VIM | 56.63 | 74.25 | 43 | 1.25 | 0.0011 |
| P30101 | Protein disulfide-isomerase A3 | PDIA3 | 55.81 | 67.13 | 41 | 1.14 | 0.0092 |
| P13639 | Elongation factor 2 | EEF2 | 61.32 | 48.60 | 35 | 1.26 | 0.0003 |
| P10809 | 60 kDa heat shock protein, mitochondrial | HSPD1 | 57.73 | 63.70 | 35 | 1.12 | 0.0416 |
| P02545 | Lamin-A/C | LMNA | 53.51 | 66.11 | 35 | 0.70 | 0.0000 |
| P22626 | Heterogeneous nuclear ribonucleoproteins A2/B1 | HNRNPA2B1 | 40.82 | 59.77 | 35 | 0.76 | 0.0054 |
| P14618 | Pyruvate kinase isozymes M1/M2 | PKM2 | 55.6 | 71.37 | 34 | 0.66 | 0.0000 |
| P26038 | Moesin | MSN | 50.29 | 65.16 | 33 | 0.71 | 0.0001 |
| P05787 | Keratin, type II cyt | KRT8 | 50.06 | 61.70 | 33 | 0.84 | 0.0055 |
| Q14204 | Cytoplasmic dynein 1 heavy chain 1 | DYNC1H1 | 63.59 | 25.59 | 32 | 1.19 | 0.0000 |
| P38646 | Stress-70 protein, mitochondrial | HSPA9 | 54.7 | 56.55 | 32 | 1.23 | 0.0048 |
| P29401 | Transketolase | TKT | 38.18 | 45.10 | 32 | 0.87 | 0.0317 |
| P08758 | Annexin A5 | ANXA5 | 39.34 | 80.31 | 30 | 0.74 | 0.0473 |
| P08729 | Keratin, type II cyt | KRT7 | 39.18 | 71.64 | 26 | 1.13 | 0.0127 |
| O43707 | Alpha-actinin-4 | ACTN4 | 38.78 | 35.35 | 26 | 0.62 | 0.0050 |
| P31327 | Carbamoyl-ph | CPS1 | 43.8 | 26.20 | 25 | 0.84 | 0.0133 |
| P00338 | L-lactate dehydrogenase A chain | LDHA | 39.13 | 63.86 | 22 | 0.76 | 0.0002 |
| P63104 | 14-3-3 protein zeta/delta | YWHAZ | 22.47 | 70.61 | 22 | 0.83 | 0.0062 |
| P27824 | Calnexin | CANX | 42.49 | 39.02 | 20 | 0.73 | 0.0228 |
| Q9Y490 | Talin-1 | TLN1 | 41.3 | 26.09 | 19 | 0.87 | 0.0239 |
| P07237 | Protein disulfide-isomerase | P4HB | 34.72 | 56.50 | 19 | 0.81 | 0.0013 |
| P10412 | Histone H1.4 | HIST1H1E | 25.35 | 72.60 | 19 | 0.50 | 0.0305 |
| P62937 | Peptidyl-prolylcis-trans isomerase A | PPIA | 30.89 | 84.24 | 18 | 0.57 | 0.0001 |
| Q06830 | Peroxiredoxin-1 | PRDX1 | 22.36 | 57.79 | 18 | 0.73 | 0.0283 |
| Q16658 | Fascin | FSCN1 | 30.63 | 57.00 | 17 | 0.73 | 0.0013 |
| P58107 | Epiplakin | EPPK1 | 26.42 | 34.79 | 17 | 1.27 | 0.0002 |
| P06744 | Gluc | GPI | 25.33 | 42.47 | 17 | 0.68 | 0.0000 |
| P16403 | Histone H1.2 | HIST1H1C | 2.79 | 62.91 | 17 | 0.42 | 0.0022 |
| Q00839 | Heterogeneous nuclear ribonucleoprotein U | HNRNPU | 29.18 | 41.09 | 16 | 0.83 | 0.0002 |
| P21796 | Voltage-dependent anion-selective channel protein 1 | VDAC1 | 23.6 | 68.55 | 16 | 1.25 | 0.0012 |
| P61978-3 | Isoform 3 of Heterogeneous nuclear ribonucleoprotein K | HNRNPK | 26.57 | 51.36 | 15 | 0.82 | 0.0390 |
| P26641 | Elongation factor 1-gamma | EEF1G | 20.43 | 40.05 | 15 | 0.79 | 0.0500 |
| Q05639 | Elongation factor 1-alpha 2 | EEF1A2 | 7.07 | 55.94 | 15 | 2.34 | 0.0097 |
| Q99832 | T-complex protein 1 subunit eta | CCT7 | 24.49 | 40.70 | 14 | 1.21 | 0.0259 |
| Q14697 | Neutral alpha-gluc | GANAB | 23.44 | 31.25 | 14 | 0.66 | 0.0017 |
| P11413 | Gluc | G6PD | 20.16 | 42.72 | 14 | 0.72 | 0.0122 |
| P07910-2 | Isoform C1 of Heterogeneous nuclear ribonucleoproteins C1/C2 | HNRNPC | 17.58 | 42.32 | 14 | 0.74 | 0.0345 |
| P15311 | Ezrin | EZR | 11.1 | 41.13 | 14 | 0.78 | 0.0168 |
| P19338 | Nucleolin | NCL | 30.5 | 37.75 | 13 | 1.44 | 0.0000 |
| Q9NZM1 | Myoferlin | MYOF | 28.54 | 28.24 | 13 | 0.74 | 0.0000 |
| P49327 | Fatty acid synthase | FASN | 28.43 | 22.10 | 13 | 1.17 | 0.0068 |
| P80723 | Brain acid soluble protein 1 | BASP1 | 26 | 84.58 | 13 | 0.35 | 0.0000 |
| Q9Y4L1 | Hypoxia up-regulated protein 1 | HYOU1 | 24.11 | 27.83 | 13 | 0.74 | 0.0212 |
| P30626 | Sorcin | SRI | 22.9 | 62.12 | 13 | 3.20 | 0.0001 |
| P26639 | Threonyl-tRNAsynthetase, cytoplasmic | TARS | 20.69 | 25.86 | 13 | 0.65 | 0.0086 |
| P07814 | Bifunctionalaminoacyl-tRNAsynthetase | EPRS | 25.14 | 23.68 | 12 | 0.86 | 0.0361 |
| P02786 | Transferrin receptor protein 1 | TFRC | 24.6 | 29.47 | 12 | 1.67 | 0.0000 |
| P23246 | Splicing factor, proline- and glutamine-rich | SFPQ | 21.02 | 39.60 | 12 | 0.87 | 0.0405 |
| P04792 | Heat shock protein beta-1 | HSPB1 | 20.96 | 61.46 | 12 | 1.81 | 0.0108 |
| P00367 | Glutamate dehydrogenase 1, mitochondrial | GLUD1 | 19.56 | 28.32 | 11 | 0.79 | 0.0234 |
| Q13263 | Transcription intermediary factor 1-beta | TRIM28 | 19.36 | 23.23 | 11 | 0.79 | 0.0026 |
| O43242 | 26S proteasome non-ATPase regulatory subunit 3 | PSMD3 | 16.94 | 21.35 | 11 | 1.37 | 0.0257 |
| Q01105 | Protein SET | SET | 15.73 | 49.66 | 11 | 0.87 | 0.0417 |
| P50395 | Rab GDP dissociation inhibitor beta | GDI2 | 15.43 | 39.33 | 11 | 0.83 | 0.0275 |
| P09874 | Poly [ADP-rib | PARP1 | 23.62 | 29.39 | 10 | 0.82 | 0.0046 |
| Q13435 | Splicing factor 3B subunit 2 | SF3B2 | 16.76 | 25.70 | 10 | 0.83 | 0.0333 |
| P31040 | Succinate dehydrogenase [ubiquinone] flavoprotein subunit, mitochondrial | SDHA | 14.45 | 29.37 | 10 | 0.73 | 0.0194 |
| P49748 | Very long-chain specific acyl-CoA dehydrogenase, mitochondrial | ACADVL | 16.89 | 26.87 | 9 | 0.84 | 0.0220 |
| P22234 | Multifunctional protein ADE2 | PAICS | 15.12 | 30.12 | 8 | 1.28 | 0.0265 |
| P62158 | Calmodulin | CALM1 | 13.72 | 71.81 | 8 | 0.75 | 0.0397 |
| O00299 | Chloride intracellular channel protein 1 | CLIC1 | 12.83 | 55.60 | 8 | 0.80 | 0.0278 |
| P15328 | Folate receptor alpha | FOLR1 | 11.98 | 29.96 | 8 | 0.16 | 0.0006 |
| Q15365 | Poly(rC)-binding protein 1 | PCBP1 | 11.53 | 44.38 | 8 | 0.85 | 0.0328 |
| O00232 | 26S proteasome non-ATPase regulatory subunit 12 | PSMD12 | 10.81 | 22.59 | 8 | 1.19 | 0.0277 |
| P55809 | Succinyl-CoA:3-ketoacid-coenzyme A transferase 1, mitochondrial | OXCT1 | 9.32 | 26.92 | 8 | 0.70 | 0.0026 |
| P20810-7 | Isoform 7 of Calpastatin | CAST | 14.88 | 28.89 | 7 | 0.60 | 0.0283 |
| P21399 | Cytoplasmic aconitatehydratase | ACO1 | 14.11 | 21.48 | 7 | 0.79 | 0.0166 |
| Q99460 | 26S proteasome non-ATPase regulatory subunit 1 | PSMD1 | 13.28 | 17.00 | 7 | 1.37 | 0.0343 |
| P30044 | Peroxiredoxin-5, mitochondrial | PRDX5 | 11.65 | 36.92 | 7 | 0.77 | 0.0382 |
| P17931 | Galectin-3 | LGALS3 | 10.31 | 31.60 | 7 | 1.43 | 0.0373 |
| P45880 | Voltage-dependent anion-selective channel protein 2 | VDAC2 | 9.97 | 39.80 | 7 | 0.77 | 0.0188 |
| P35998 | 26S protease regulatory subunit 7 | PSMC2 | 14.25 | 38.80 | 6 | 1.22 | 0.0145 |
| Q9Y2W1 | Thyroid hormone receptor-associated protein 3 | THRAP3 | 13.03 | 22.72 | 6 | 0.71 | 0.0196 |
| P41250 | Glycyl-tRNAsynthetase | GARS | 12.13 | 18.67 | 6 | 0.70 | 0.0014 |
| Q07065 | Cyt | CKAP4 | 12.05 | 27.24 | 6 | 0.77 | 0.0389 |
| P08183 | Multidrug resistance protein 1 | ABCB1 | 11.82 | 20.39 | 6 | 2.31 | 0.0056 |
| P99999 | Cytochrome c | CYCS | 10.43 | 60.00 | 6 | 0.67 | 0.0271 |
| P47756-2 | Isoform 2 of F-actin-capping protein subunit beta | CAPZB | 9.69 | 32.35 | 6 | 0.75 | 0.0169 |
| P12277 | Creatine kinase B-type | CKB | 9.23 | 25.72 | 6 | 1.50 | 0.0012 |
| P62328 | Thym | TMSB4X | 8.54 | 77.27 | 6 | 0.57 | 0.0250 |
| P13667 | Protein disulfide-isomerase A4 | PDIA4 | 11.98 | 30.70 | 5 | 0.78 | 0.0021 |
| O43399 | Tumor protein D54 | TPD52L2 | 11.07 | 38.35 | 5 | 1.23 | 0.0179 |
| Q9UBE0 | SUMO-activating enzyme subunit 1 | SAE1 | 10.19 | 30.06 | 5 | 0.70 | 0.0231 |
| Q15056 | Eukaryotic translation initiation factor 4H | EIF4H | 9.7 | 38.71 | 5 | 0.58 | 0.0006 |
| Q14839 | Chromodomain-helicase-DNA-binding protein 4 | CHD4 | 9.57 | 17.42 | 5 | 0.89 | 0.0440 |
| Q9UHB9 | Signal recognition particle 68 kDa protein | SRP68 | 9.18 | 24.72 | 5 | 1.26 | 0.0086 |
| Q7L576 | Cytoplasmic FMR1-interacting protein 1 | CYFIP1 | 8.98 | 13.65 | 5 | 1.20 | 0.0492 |
| Q13200 | 26S proteasome non-ATPase regulatory subunit 2 | PSMD2 | 11.52 | 24.45 | 4 | 1.31 | 0.0047 |
| Q9BQG0 | Myb-binding protein 1A | MYBBP1A | 10.48 | 17.24 | 4 | 1.34 | 0.0354 |
| Q9UHB6 | LIM domain and actin-binding protein 1 | LIMA1 | 9.91 | 24.51 | 4 | 1.88 | 0.0001 |
| Q9H0A0 | N-acetyltransferase 10 | NAT10 | 9.41 | 16.88 | 4 | 1.31 | 0.0146 |
| Q9Y5B9 | FACT complex subunit SPT16 | SUPT16H | 8.86 | 21.01 | 4 | 0.78 | 0.0465 |
| O75531 | Barrier-to-autointegration factor | BANF1 | 8.59 | 75.28 | 4 | 0.56 | 0.0050 |
| P16104 | Histone H2A.x | H2AFX | 8.26 | 40.56 | 4 | 0.58 | 0.0138 |
| Q13619 | Cullin-4A | CUL4A | 7.44 | 18.31 | 4 | 2.01 | 0.0181 |
| P09960 | Leukotriene A-4 hydrolase | LTA4H | 7.32 | 12.77 | 4 | 1.43 | 0.0092 |
| O43175 | D-3-ph | PHGDH | 7.31 | 19.14 | 4 | 2.34 | 0.0359 |
| P50995 | Annexin A11 | ANXA11 | 7.13 | 24.95 | 4 | 0.61 | 0.0228 |
| Q96TA1 | Niban-like protein 1 | FAM129B | 6.77 | 19.51 | 4 | 0.83 | 0.0404 |
| O75367 | Core histone macro-H2A.1 | H2AFY | 6.19 | 22.04 | 4 | 0.64 | 0.0388 |
| Q71DI3 | Histone H3.2 | HIST2H3A | 3.94 | 55.88 | 4 | 0.45 | 0.0000 |
| Q13423 | NAD(P) transhydrogenase, mitochondrial | NNT | 7.21 | 11.33 | 3 | 0.55 | 0.0010 |
| P04181 | Ornithine aminotransferase, mitochondrial | OAT | 6.94 | 26.65 | 3 | 1.52 | 0.0142 |
| P30085 | UMP-CMP kinase | CMPK1 | 6.94 | 35.20 | 3 | 0.71 | 0.0158 |
| Q99497 | Protein DJ-1 | PARK7 | 6.86 | 49.21 | 3 | 0.71 | 0.0396 |
| Q10471 | Polypeptide N-acetylgalact | GALNT2 | 6.67 | 20.49 | 3 | 0.59 | 0.0118 |
| Q15459 | Splicing factor 3A subunit 1 | SF3A1 | 6.57 | 16.39 | 3 | 0.79 | 0.0142 |
| Q05682 | Caldesmon | CALD1 | 6.43 | 22.57 | 3 | 0.41 | 0.0359 |
| Q13228 | Selenium-binding protein 1 | SELENBP1 | 6.2 | 18.43 | 3 | 0.62 | 0.0103 |
| P78347 | General transcription factor II-I | GTF2I | 5.89 | 16.03 | 3 | 0.56 | 0.0030 |
| Q13724 | Mann | MOGS | 5.04 | 10.75 | 3 | 1.56 | 0.0085 |
| P46934-4 | Isoform 4 of E3 ubiquitin-protein ligase NEDD4 | NEDD4 | 5.04 | 15.67 | 3 | 1.30 | 0.0180 |
| P09497 | Clathrin light chain B | CLTB | 4.94 | 29.26 | 2 | 0.66 | 0.0176 |
| Q13428 | Treacle protein | TCOF1 | 4.57 | 9.75 | 2 | 0.68 | 0.0080 |
| P17096 | High mobility group protein HMG-I/HMG-Y | HMGA1 | 4.43 | 38.32 | 2 | 0.52 | 0.0076 |
| Q8WW12 | PEST proteolytic signal-containing nuclear protein | PCNP | 4.41 | 26.97 | 2 | 0.60 | 0.0343 |
| P48637 | Glutathione synthetase | GSS | 4.38 | 25.74 | 2 | 1.38 | 0.0242 |
| P31350 | Ribonucle | RRM2 | 3.92 | 19.54 | 2 | 1.47 | 0.0146 |
| Q96D15 | Reticulocalbin-3 | RCN3 | 3.87 | 25.91 | 2 | 0.63 | 0.0111 |
| Q9NZL4 | Hsp70-binding protein 1 | HSPBP1 | 3.62 | 12.71 | 2 | 0.70 | 0.0345 |
| Q9UDY2 | Tight junction protein ZO-2 | TJP2 | 3.61 | 18.24 | 2 | 0.80 | 0.0274 |
| P59998 | Actin-related protein 2/3 complex subunit 4 | ARPC4 | 3.57 | 25.60 | 2 | 1.51 | 0.0455 |
| P58546 | Myotrophin | MTPN | 3.56 | 33.05 | 2 | 0.66 | 0.0451 |
| Q9H3P7 | Golgi resident protein GCP60 | ACBD3 | 3.4 | 15.34 | 2 | 0.76 | 0.0374 |
| Q9UK76 | Hematological and neurological expressed 1 protein | HN1 | 3.36 | 40.26 | 2 | 1.79 | 0.0240 |

^a^Protein samples were labeled with 113 for SGC7901 and with 114 for SGC7901/ADR.

**Table 4. Differentially expressed proteins in SGC7901/VCR cells compared with SGC7901 cells identified by iTRAQ-MS/MS**

| **Accession** | **Protein Name** | **Gene Symbol** | **Unused** | **%Cov** | **Pepti-des**  **(95%)** | **115:113^a^** | **Pvalue** |
| --- | --- | --- | --- | --- | --- | --- | --- |
| P21333 | Filamin-A | FLNA | 161.52 | 49.45 | 105 | 1.20 | 0.0000 |
| P35579 | Myosin-9 | MYH9 | 105.29 | 41.38 | 53 | 1.12 | 0.0003 |
| Q15149-8 | Isoform Plectin-3 of Plectin-1 | PLEC1 | 97.12 | 38.03 | 48 | 0.92 | 0.0145 |
| P06733 | Alpha-enolase | ENO1 | 90.45 | 86.41 | 76 | 0.79 | 0.0002 |
| P11021 | 78 kDa glucose-regulated protein | HSPA5 | 84.31 | 59.94 | 58 | 0.74 | 0.0000 |
| Q00610-2 | Isoform 2 of Clathrin heavy chain 1 | CLTC | 70 | 43.99 | 40 | 0.85 | 0.0002 |
| Q09666 | Neuroblast differentiation-associated protein AHNAK | AHNAK | 66.13 | 46.37 | 43 | 0.92 | 0.0073 |
| Q14204 | Cytoplasmic dynein 1 heavy chain 1 | DYNC1H1 | 63.59 | 25.59 | 32 | 1.12 | 0.0000 |
| P06576 | ATP synthase subunit beta, mitochondrial | ATP5B | 59.03 | 58.60 | 38 | 0.84 | 0.0022 |
| P04406 | Glyceraldehyde-3-phosphate dehydrogenase | GAPDH | 58.58 | 71.34 | 53 | 0.62 | 0.0000 |
| P07355 | Annexin A2 | ANXA2 | 55.67 | 71.09 | 41 | 1.12 | 0.0120 |
| P14618 | Pyruvate kinase isozymes M1/M2 | PKM2 | 55.6 | 71.37 | 34 | 0.70 | 0.0000 |
| P38646 | Stress-70 protein, mitochondrial | HSPA9 | 54.7 | 56.55 | 32 | 1.15 | 0.0325 |
| P02545 | Lamin-A/C | LMNA | 53.51 | 66.11 | 35 | 0.74 | 0.0000 |
| P00558 | Phosphoglycerate kinase 1 | PGK1 | 51.88 | 70.26 | 33 | 0.92 | 0.0466 |
| P26038 | Moesin | MSN | 50.29 | 65.16 | 33 | 0.83 | 0.0000 |
| P05787 | Keratin, type II cytoskeletal 8 | KRT8 | 50.06 | 61.70 | 33 | 0.74 | 0.0000 |
| P31327 | Carbamoyl-phosphate synthase [ammonia], mitochondrial | CPS1 | 43.8 | 26.20 | 25 | 0.63 | 0.0000 |
| P55072 | Transitional endoplasmic reticulum ATPase | VCP | 43.36 | 54.09 | 28 | 0.89 | 0.0084 |
| P27824 | Calnexin | CANX | 42.49 | 39.02 | 20 | 0.76 | 0.0071 |
| P04075 | Fructose-bisphosphate aldolase A | ALDOA | 41.97 | 76.92 | 33 | 0.81 | 0.0007 |
| P22626 | Heterogeneous nuclear ribonucleoproteins A2/B1 | HNRNPA2B1 | 40.82 | 59.77 | 35 | 0.72 | 0.0000 |
| P08758 | Annexin A5 | ANXA5 | 39.34 | 80.31 | 30 | 0.73 | 0.0000 |
| P00338 | L-lactate dehydrogenase A chain | LDHA | 39.13 | 63.86 | 22 | 0.68 | 0.0000 |
| O43707 | Alpha-actinin-4 | ACTN4 | 38.78 | 35.35 | 26 | 0.80 | 0.0039 |
| P08107 | Heat shock 70 kDa protein 1A/1B | HSPA1A | 37.96 | 62.71 | 50 | 1.22 | 0.0262 |
| P06748 | Nucleophosmin | NPM1 | 36.66 | 61.90 | 37 | 1.27 | 0.0035 |
| P05783 | Keratin, type I cytoskeletal 18 | KRT18 | 36.09 | 69.77 | 23 | 0.84 | 0.0000 |
| P07237 | Protein disulfide-isomerase | P4HB | 34.72 | 56.50 | 19 | 0.69 | 0.0000 |
| O00571 | ATP-dependent RNA helicase DDX3X | DDX3X | 32.6 | 42.90 | 17 | 1.12 | 0.0271 |
| P25705 | ATP synthase subunit alpha, mitochondrial | ATP5A1 | 32.59 | 58.59 | 21 | 0.89 | 0.0022 |
| P78371 | T-complex protein 1 subunit beta | CCT2 | 32.1 | 57.94 | 20 | 1.10 | 0.0446 |
| P62937 | Peptidyl-prolyl cis-trans isomerase A | PPIA | 30.89 | 84.24 | 18 | 0.41 | 0.0000 |
| Q16658 | Fascin | FSCN1 | 30.63 | 57.00 | 17 | 0.83 | 0.0032 |
| P19338 | Nucleolin | NCL | 30.5 | 37.75 | 13 | 1.63 | 0.0000 |
| P37802 | Transgelin-2 | TAGLN2 | 30.39 | 79.40 | 21 | 0.81 | 0.0023 |
| P40926 | Malate dehydrogenase, mitochondrial | MDH2 | 30.23 | 60.36 | 20 | 0.79 | 0.0072 |
| P50990 | T-complex protein 1 subunit theta | CCT8 | 29.53 | 42.15 | 16 | 1.10 | 0.0198 |
| Q00839 | Heterogeneous nuclear ribonucleoprotein U | HNRNPU | 29.18 | 41.09 | 16 | 0.88 | 0.0133 |
| P62258 | 14-3-3 protein epsilon | YWHAE | 28.64 | 72.16 | 23 | 0.85 | 0.0062 |
| Q9NZM1 | Myoferlin | MYOF | 28.54 | 28.24 | 13 | 0.79 | 0.0000 |
| P78527-2 | Isoform 2 of DNA-dependent protein kinase catalytic subunit | PRKDC | 26.31 | 17.50 | 11 | 0.82 | 0.0003 |
| P13797 | Plastin-3 | PLS3 | 26.07 | 36.19 | 13 | 1.12 | 0.0190 |
| P80723 | Brain acid soluble protein 1 | BASP1 | 26 | 84.58 | 13 | 0.33 | 0.0000 |
| P00966 | Argininosuccinate synthase | ASS1 | 25.56 | 42.48 | 15 | 0.75 | 0.0002 |
| P10412 | Histone H1.4 | HIST1H1E | 25.35 | 72.60 | 19 | 0.60 | 0.0295 |
| P06744 | Glucose-6-phosphate isomerase | GPI | 25.33 | 42.47 | 17 | 0.62 | 0.0000 |
| P07814 | Bifunctional aminoacyl-tRNA synthetase | EPRS | 25.14 | 23.68 | 12 | 0.84 | 0.0008 |
| P02786 | Transferrin receptor protein 1 | TFRC | 24.6 | 29.47 | 12 | 1.11 | 0.0429 |
| Q99832 | T-complex protein 1 subunit eta | CCT7 | 24.49 | 40.70 | 14 | 1.22 | 0.0192 |
| Q14152 | Eukaryotic translation initiation factor 3 subunit A | EIF3A | 24.23 | 35.82 | 13 | 1.09 | 0.0187 |
| P53621 | Coatomer subunit alpha | COPA | 24.17 | 29.66 | 13 | 0.76 | 0.0001 |
| Q9Y4L1 | Hypoxia up-regulated protein 1 | HYOU1 | 24.11 | 27.83 | 13 | 0.73 | 0.0010 |
| P67936 | Tropomyosin alpha-4 chain | TPM4 | 23.85 | 62.90 | 11 | 1.18 | 0.0384 |
| Q14697 | Neutral alpha-glucosidase AB | GANAB | 23.44 | 31.25 | 14 | 0.74 | 0.0261 |
| P30626 | Sorcin | SRI | 22.9 | 62.12 | 13 | 2.44 | 0.0003 |
| Q06830 | Peroxiredoxin-1 | PRDX1 | 22.36 | 57.79 | 18 | 0.78 | 0.0038 |
| P62805 | Histone H4 | HIST1H4A | 22.22 | 70.87 | 29 | 0.75 | 0.0397 |
| P27797 | Calreticulin | CALR | 21.85 | 42.93 | 12 | 0.84 | 0.0077 |
| Q15233 | Non-POU domain-containing octamer-binding protein | NONO | 21.44 | 43.31 | 13 | 0.82 | 0.0470 |
| Q15084 | Protein disulfide-isomerase A6 | PDIA6 | 21.25 | 36.36 | 14 | 0.91 | 0.0436 |
| Q04695 | Keratin, type I cytoskeletal 17 | KRT17 | 21.19 | 33.80 | 12 | 0.66 | 0.0000 |
| P23246 | Splicing factor, proline- and glutamine-rich | SFPQ | 21.02 | 39.60 | 12 | 0.86 | 0.0143 |
| P09211 | Glutathione S-transferase P | GSTP1 | 20.49 | 66.19 | 24 | 0.71 | 0.0002 |
| P26641 | Elongation factor 1-gamma | EEF1G | 20.43 | 40.05 | 15 | 0.66 | 0.0007 |
| P00367 | Glutamate dehydrogenase 1, mitochondrial | GLUD1 | 19.56 | 28.32 | 11 | 0.75 | 0.0024 |
| P08195 | 4F2 cell-surface antigen heavy chain | SLC3A2 | 19.25 | 32.70 | 12 | 0.74 | 0.0000 |
| P00505 | Aspartate aminotransferase, mitochondrial | GOT2 | 19.07 | 42.09 | 12 | 1.14 | 0.0193 |
| P12814 | Alpha-actinin-1 | ACTN1 | 18.3 | 35.76 | 24 | 1.49 | 0.0007 |
| Q99613 | Eukaryotic translation initiation factor 3 subunit C | EIF3C | 18.06 | 29.79 | 9 | 1.15 | 0.0335 |
| P07910-2 | Isoform C1 of Heterogeneous nuclear ribonucleoproteins C1/C2 | HNRNPC | 17.58 | 42.32 | 14 | 0.86 | 0.0328 |
| P09429 | High mobility group protein B1 | HMGB1 | 17.57 | 49.30 | 11 | 0.82 | 0.0386 |
| P49748 | Very long-chain specific acyl-CoA dehydrogenase, mitochondrial | ACADVL | 16.89 | 26.87 | 9 | 0.76 | 0.0008 |
| P33992 | DNA replication licensing factor MCM5 | MCM5 | 16.78 | 30.11 | 9 | 0.76 | 0.0094 |
| P23284 | Peptidyl-prolyl cis-trans isomerase B | PPIB | 16.21 | 62.96 | 8 | 0.76 | 0.0146 |
| P50395 | Rab GDP dissociation inhibitor beta | GDI2 | 15.43 | 39.33 | 11 | 0.82 | 0.0195 |
| Q14566 | DNA replication licensing factor MCM6 | MCM6 | 15.36 | 21.80 | 8 | 0.80 | 0.0141 |
| P55209 | Nucleosome assembly protein 1-like 1 | NAP1L1 | 15.27 | 29.41 | 10 | 1.37 | 0.0245 |
| O60664 | Perilipin-3 | PLIN3 | 14.94 | 35.02 | 7 | 1.35 | 0.0048 |
| P20810-7 | Isoform 7 of Calpastatin | CAST | 14.88 | 28.89 | 7 | 0.59 | 0.0008 |
| Q01813 | 6-phosphofructokinase type C | PFKP | 14.87 | 22.96 | 8 | 1.48 | 0.0013 |
| Q9UNF1 | Melanoma-associated antigen D2 | MAGED2 | 14.81 | 25.74 | 9 | 0.74 | 0.0235 |
| O00429 | Dynamin-1-like protein | DNM1L | 14.01 | 24.05 | 7 | 1.18 | 0.0306 |
| P38606 | V-type proton ATPase catalytic subunit A | ATP6V1A | 13.06 | 29.17 | 8 | 0.84 | 0.0146 |
| Q14315 | Filamin-C | FLNC | 12.97 | 22.39 | 16 | 1.25 | 0.0393 |
| P33993 | DNA replication licensing factor MCM7 | MCM7 | 12.97 | 31.85 | 7 | 0.78 | 0.0171 |
| P48735 | Isocitrate dehydrogenase [NADP], mitochondrial | IDH2 | 12.71 | 33.85 | 6 | 0.80 | 0.0213 |
| P41250 | Glycyl-tRNA synthetase | GARS | 12.13 | 18.67 | 6 | 0.79 | 0.0203 |
| Q07065 | Cytoskeleton-associated protein 4 | CKAP4 | 12.05 | 27.24 | 6 | 0.86 | 0.0308 |
| P13667 | Protein disulfide-isomerase A4 | PDIA4 | 11.98 | 30.70 | 5 | 0.82 | 0.0028 |
| P15328 | Folate receptor alpha | FOLR1 | 11.98 | 29.96 | 8 | 0.14 | 0.0006 |
| Q6XQN6-2 | Isoform 2 of Nicotinate phosphoribosyltransferase | NAPRT1 | 11.86 | 26.28 | 7 | 0.58 | 0.0058 |
| P08183 | Multidrug resistance protein 1 | ABCB1 | 11.82 | 20.39 | 6 | 2.78 | 0.0036 |
| P33176 | Kinesin-1 heavy chain | KIF5B | 11.66 | 24.82 | 6 | 1.22 | 0.0066 |
| P35606 | Coatomer subunit beta' | COPB2 | 11.63 | 34.00 | 6 | 0.79 | 0.0107 |
| Q15942 | Zyxin | ZYX | 11.58 | 32.52 | 7 | 1.35 | 0.0077 |
| Q13200 | 26S proteasome non-ATPase regulatory subunit 2 | PSMD2 | 11.52 | 24.45 | 4 | 1.31 | 0.0066 |
| P48444 | Coatomer subunit delta | ARCN1 | 11.36 | 22.70 | 6 | 0.76 | 0.0103 |
| P49591 | Seryl-tRNA synthetase, cytoplasmic | SARS | 11.31 | 18.87 | 5 | 0.82 | 0.0178 |
| P36871 | Phosphoglucomutase-1 | PGM1 | 11.19 | 26.33 | 6 | 0.78 | 0.0442 |
| O43399 | Tumor protein D54 | TPD52L2 | 11.07 | 38.35 | 5 | 1.35 | 0.0049 |
| P63241-2 | Isoform A of Eukaryotic translation initiation factor 5A-1 | EIF5A | 11 | 45.65 | 9 | 0.78 | 0.0457 |
| Q06210 | Glucosamine--fructose-6-phosphate aminotransferase [isomerizing] 1 | GFPT1 | 10.96 | 29.33 | 5 | 0.77 | 0.0180 |
| P46781 | 40S ribosomal protein S9 | RPS9 | 10.88 | 52.58 | 6 | 0.83 | 0.0170 |
| P10599 | Thioredoxin | TXN | 10.8 | 51.43 | 6 | 0.66 | 0.0162 |
| Q13185 | Chromobox protein homolog 3 | CBX3 | 10.58 | 44.26 | 5 | 0.76 | 0.0315 |
| Q02790 | Peptidyl-prolyl cis-trans isomerase FKBP4 | FKBP4 | 10.5 | 35.29 | 5 | 1.21 | 0.0047 |
| Q9BQG0 | Myb-binding protein 1A | MYBBP1A | 10.48 | 17.24 | 4 | 1.34 | 0.0148 |
| P99999 | Cytochrome c | CYCS | 10.43 | 60.00 | 6 | 0.63 | 0.0033 |
| P17931 | Galectin-3 | LGALS3 | 10.31 | 31.60 | 7 | 0.75 | 0.0119 |
| P32119 | Peroxiredoxin-2 | PRDX2 | 10.21 | 43.43 | 10 | 0.76 | 0.0057 |
| Q9UBE0 | SUMO-activating enzyme subunit 1 | SAE1 | 10.19 | 30.06 | 5 | 0.82 | 0.0438 |
| Q99829 | Copine-1 | CPNE1 | 10.17 | 17.32 | 6 | 1.32 | 0.0370 |
| P12268 | Inosine-5'-monophosphate dehydrogenase 2 | IMPDH2 | 9.93 | 26.07 | 4 | 1.42 | 0.0007 |
| Q9UHB6 | LIM domain and actin-binding protein 1 | LIMA1 | 9.91 | 24.51 | 4 | 2.70 | 0.0001 |
| P09382 | Galectin-1 | LGALS1 | 9.83 | 57.04 | 9 | 0.82 | 0.0196 |
| O75340 | Programmed cell death protein 6 | PDCD6 | 9.77 | 52.36 | 5 | 0.66 | 0.0046 |
| Q15056 | Eukaryotic translation initiation factor 4H | EIF4H | 9.7 | 38.71 | 5 | 0.78 | 0.0181 |
| P17812 | CTP synthase 1 | CTPS | 9.67 | 24.87 | 7 | 1.41 | 0.0026 |
| Q9NYU2 | UDP-glucose:glycoprotein glucosyltransferase 1 | UGGT1 | 9.54 | 14.98 | 5 | 0.73 | 0.0012 |
| O43852 | Calumenin | CALU | 9.19 | 31.75 | 4 | 0.65 | 0.0211 |
| O75396 | Vesicle-trafficking protein SEC22b | SEC22B | 9.12 | 39.53 | 6 | 0.70 | 0.0429 |
| Q14019 | Coactosin-like protein | COTL1 | 8.97 | 72.54 | 4 | 1.28 | 0.0108 |
| P53999 | Activated RNA polymerase II transcriptional coactivator p15 | SUB1 | 8.72 | 49.61 | 4 | 0.68 | 0.0026 |
| O75531 | Barrier-to-autointegration factor | BANF1 | 8.59 | 75.28 | 4 | 0.73 | 0.0099 |
| P16401 | Histone H1.5 | HIST1H1B | 8.42 | 63.72 | 11 | 0.71 | 0.0346 |
| O00461 | Golgi integral membrane protein 4 | GOLIM4 | 8.21 | 18.10 | 4 | 0.67 | 0.0447 |
| Q16698 | 2,4-dienoyl-CoA reductase, mitochondrial | DECR1 | 8.2 | 21.79 | 4 | 0.60 | 0.0111 |
| Q13011 | Delta(3,5)-Delta(2,4)-dienoyl-CoA isomerase, mitochondrial | ECH1 | 8.14 | 21.95 | 4 | 0.61 | 0.0104 |
| Q13045 | Protein flightless-1 homolog | FLII | 8.09 | 18.75 | 4 | 1.22 | 0.0349 |
| Q9Y678 | Coatomer subunit gamma | COPG | 8.07 | 17.05 | 4 | 0.79 | 0.0439 |
| P61981 | 14-3-3 protein gamma | YWHAG | 7.98 | 49.80 | 14 | 0.82 | 0.0470 |
| P20042 | Eukaryotic translation initiation factor 2 subunit 2 | EIF2S2 | 7.91 | 33.33 | 4 | 1.37 | 0.0275 |
| P12004 | Proliferating cell nuclear antigen | PCNA | 7.46 | 45.59 | 4 | 0.77 | 0.0054 |
| P16070 | CD44 antigen | CD44 | 7.39 | 9.30 | 4 | 1.45 | 0.0427 |
| Q9NQC3-2 | Isoform Foocen-M of Reticulon-4 | RTN4 | 7.37 | 27.88 | 4 | 1.29 | 0.0288 |
| O43175 | D-3-phosphoglycerate dehydrogenase | PHGDH | 7.31 | 19.14 | 4 | 2.22 | 0.0297 |
| Q13423 | NAD(P) transhydrogenase, mitochondrial | NNT | 7.21 | 11.33 | 3 | 0.63 | 0.0020 |
| P50995 | Annexin A11 | ANXA11 | 7.13 | 24.95 | 4 | 0.50 | 0.0025 |
| P83731 | 60S ribosomal protein L24 | RPL24 | 6.97 | 33.76 | 5 | 0.83 | 0.0469 |
| O43847 | Nardilysin | NRD1 | 6.95 | 12.17 | 4 | 1.27 | 0.0193 |
| P26447 | Protein S100-A4 | S100A4 | 6.73 | 36.63 | 4 | 0.40 | 0.0019 |
| Q10471 | Polypeptide N-acetylgalactosaminyltransferase 2 | GALNT2 | 6.67 | 20.49 | 3 | 0.61 | 0.0016 |
| P11498 | Pyruvate carboxylase, mitochondrial | PC | 6.36 | 19.61 | 4 | 0.73 | 0.0192 |
| P61160 | Actin-related protein 2 | ACTR2 | 6.24 | 24.11 | 3 | 1.31 | 0.0305 |
| Q13228 | Selenium-binding protein 1 | SELENBP1 | 6.2 | 18.43 | 3 | 0.66 | 0.0158 |
| P49257 | Protein ERGIC-53 | LMAN1 | 6.1 | 22.16 | 3 | 0.67 | 0.0174 |
| P78347 | General transcription factor II-I | GTF2I | 5.89 | 16.03 | 3 | 0.73 | 0.0188 |
| Q9H4A4 | Aminopeptidase B | RNPEP | 5.53 | 17.69 | 5 | 0.75 | 0.0380 |
| Q9BUJ2 | Heterogeneous nuclear ribonucleoprotein U-like protein 1 | HNRNPUL1 | 5.39 | 29.91 | 3 | 0.73 | 0.0398 |
| Q969X5 | Endoplasmic reticulum-Golgi intermediate compartment protein 1 | ERGIC1 | 5.16 | 21.03 | 4 | 0.70 | 0.0168 |
| O43719 | HIV Tat-specific factor 1 | HTATSF1 | 5.05 | 14.57 | 3 | 0.82 | 0.0477 |
| O95336 | 6-phosphogluconolactonase | PGLS | 5.05 | 27.52 | 3 | 0.66 | 0.0166 |
| P46934-4 | Isoform 4 of E3 ubiquitin-protein ligase NEDD4 | NEDD4 | 5.04 | 15.67 | 3 | 1.44 | 0.0119 |
| Q6NZI2 | Polymerase I and transcript release factor | PTRF | 4.99 | 14.87 | 4 | 0.53 | 0.0214 |
| P27694 | Replication protein A 70 kDa DNA-binding subunit | RPA1 | 4.85 | 16.23 | 2 | 0.70 | 0.0022 |
| P78417 | Glutathione S-transferase omega-1 | GSTO1 | 4.64 | 19.50 | 2 | 0.71 | 0.0381 |
| P48637 | Glutathione synthetase | GSS | 4.38 | 25.74 | 2 | 1.50 | 0.0278 |
| Q9Y5M8 | Signal recognition particle receptor subunit beta | SRPRB | 4.27 | 36.90 | 2 | 0.66 | 0.0109 |
| P55265 | Double-stranded RNA-specific adenosine deaminase | ADAR | 4.25 | 9.13 | 2 | 0.80 | 0.0445 |
| P31350 | Ribonucleoside-diphosphate reductase subunit M2 | RRM2 | 3.92 | 19.54 | 2 | 1.29 | 0.0405 |
| Q96D15 | Reticulocalbin-3 | RCN3 | 3.87 | 25.91 | 2 | 0.76 | 0.0181 |
| Q9NX58 | Cell growth-regulating nucleolar protein | LYAR | 3.46 | 15.04 | 2 | 1.36 | 0.0252 |
| P16403 | Histone H1.2 | HIST1H1C | 2.79 | 62.91 | 17 | 0.56 | 0.0041 |
| P55060 | Exportin-2 | CSE1L | 20.47 | 22.66 | 12 | 1.12 | 0.0345 |

^a^Protein samples were labeled with 113 for SGC7901 and with 115 for SGC7901/VCR.

**Table 5.** ProteinRank scores for the 11 seed proteins (flagged by *) and the top 100 ranked proteins in ADR study

| **Rank** | **Gene Symbol** | **HPRD ID** | **RankScore** | **P Value** |
| --- | --- | --- | --- | --- |
| 1 | ABCB1* | 01370 | 1.1842 | 0.0000 |
| 2 | ABCB11* | 04436 | 1.1712 | 0.0001 |
| 3 | AKT1* | 01261 | 1.0594 | 0.0002 |
| 4 | BAD* | 04409 | 1.0535 | 0.0004 |
| 5 | BCL2* | 01045 | 1.0444 | 0.0003 |
| 6 | TERT* | 01754 | 1.0342 | 0.0004 |
| 7 | CYP3A4* | 00484 | 1.0055 | 0.0005 |
| 8 | TOP2A* | 00536 | 1.0051 | 0.0006 |
| 9 | ABCB8* | 10400 | 1.0046 | 0.0006 |
| 10 | ZNRD1* | 09601 | 1.0045 | 0.0010 |
| 11 | MAD2L1* | 03274 | 1.0031 | 0.0012 |
| 12 | HAX1 | 12075 | 0.9779 | 0.0012 |
| 13 | RNF2 | 07028 | 0.7204 | 0.0012 |
| 14 | PIM3 | 15137 | 0.5205 | 0.0012 |
| 15 | TPP1 | 06415 | 0.4990 | 0.0015 |
| 16 | CAV1 | 03028 | 0.4267 | 0.0017 |
| 17 | SMG6 | 06502 | 0.4097 | 0.0020 |
| 18 | DFFB | 03532 | 0.3495 | 0.0017 |
| 19 | BCL2L1 | 02497 | 0.3374 | 0.0015 |
| 20 | TP53AIP1 | 10397 | 0.3307 | 0.0021 |
| 21 | CPT1A | 02755 | 0.3307 | 0.0020 |
| 22 | SMG5 | 16869 | 0.2930 | 0.0021 |
| 23 | SAT1 | 02431 | 0.2859 | 0.0022 |
| 24 | NMB | 01210 | 0.2757 | 0.0027 |
| 25 | BNIPL | 16557 | 0.2723 | 0.0023 |
| 26 | METTL1 | 06834 | 0.2643 | 0.0028 |
| 27 | PLXNA1 | 11868 | 0.2543 | 0.0025 |
| 28 | DLC1 | 05035 | 0.2527 | 0.0029 |
| 29 | CCDC88A | 11131 | 0.2527 | 0.0027 |
| 30 | TCL6 | 05104 | 0.2527 | 0.0031 |
| 31 | THEM4 | 16215 | 0.2527 | 0.0028 |
| 32 | UXS1 | 15642 | 0.2527 | 0.0034 |
| 33 | HMOX1 | 00782 | 0.2498 | 0.0030 |
| 34 | - | 05934 | 0.2445 | 0.0030 |
| 35 | PCGF6 | 07427 | 0.2322 | 0.0032 |
| 36 | TCL1B | 04795 | 0.2271 | 0.0033 |
| 37 | SOD1 | 00937 | 0.2224 | 0.0035 |
| 38 | NCL | 01245 | 0.2105 | 0.0042 |
| 39 | KIAA0513 | 17192 | 0.2001 | 0.0048 |
| 40 | AHNAK | 14684 | 0.1941 | 0.0041 |
| 41 | NONO | 02098 | 0.1924 | 0.0039 |
| 42 | HSP90AA1 | 00777 | 0.1916 | 0.0042 |
| 43 | MYST1 | 11381 | 0.1892 | 0.0048 |
| 44 | BFAR | 16548 | 0.1838 | 0.0040 |
| 45 | ITM2B | 04878 | 0.1837 | 0.0046 |
| 46 | HRK | 04581 | 0.1782 | 0.0051 |
| 47 | ABCB4 | 01371 | 0.1772 | 0.0047 |
| 48 | PDK2 | 03955 | 0.1755 | 0.0045 |
| 49 | RAB3D | 05070 | 0.1727 | 0.0048 |
| 50 | BNIP2 | 04481 | 0.1727 | 0.0050 |
| 51 | RING1 | 03624 | 0.1704 | 0.0050 |
| 52 | RYBP | 09607 | 0.1596 | 0.0044 |
| 53 | TFCP2 | 01790 | 0.1590 | 0.0053 |
| 54 | BCL2L14 | 05841 | 0.1574 | 0.0057 |
| 55 | SPNS1 | 11599 | 0.1574 | 0.0049 |
| 56 | TMBIM6 | 02851 | 0.1568 | 0.0054 |
| 57 | PKD2 | 01437 | 0.1557 | 0.0055 |
| 58 | NMBR | 01211 | 0.1549 | 0.0059 |
| 59 | YWHAZ | 03183 | 0.1520 | 0.0055 |
| 60 | ATP2A2 | 00161 | 0.1486 | 0.0058 |
| 61 | RAF1 | 01265 | 0.1458 | 0.0064 |
| 62 | BNIP1 | 04480 | 0.1455 | 0.0056 |
| 63 | CYCS | 00479 | 0.1452 | 0.0062 |
| 64 | VEGFA | 01889 | 0.1427 | 0.0067 |
| 65 | BNIP3 | 04482 | 0.1422 | 0.0063 |
| 66 | WDR4 | 09330 | 0.1362 | 0.0065 |
| 67 | BECN1 | 05087 | 0.1347 | 0.0068 |
| 68 | SEMA6D | 10221 | 0.1308 | 0.0070 |
| 69 | SVIL | 04992 | 0.1300 | 0.0062 |
| 70 | ATXN2 | 03307 | 0.1300 | 0.0079 |
| 71 | IL1A | 00988 | 0.1297 | 0.0072 |
| 72 | BLVRB | 02967 | 0.1286 | 0.0071 |
| 73 | PHC1 | 16009 | 0.1280 | 0.0077 |
| 74 | PARP1 | 01435 | 0.1268 | 0.0074 |
| 75 | TOPBP1 | 09678 | 0.1245 | 0.0082 |
| 76 | VDAC1 | 05137 | 0.1220 | 0.0080 |
| 77 | TCL1A | 01744 | 0.1219 | 0.0080 |
| 78 | MTOR | 03134 | 0.1202 | 0.0085 |
| 79 | PPARGC1B | 10594 | 0.1200 | 0.0087 |
| 80 | BIK | 04547 | 0.1191 | 0.0083 |
| 81 | DGKD | 03492 | 0.1178 | 0.0086 |
| 82 | KDM5D | 02464 | 0.1172 | 0.0080 |
| 83 | PPP3CA | 00234 | 0.1150 | 0.0085 |
| 84 | BLK | 01866 | 0.1148 | 0.0087 |
| 85 | S1PR1 | 03578 | 0.1133 | 0.0087 |
| 86 | SUMO4 | 10584 | 0.1126 | 0.0085 |
| 87 | TP53 | 01859 | 0.1124 | 0.0086 |
| 88 | APPL1 | 05053 | 0.1122 | 0.0092 |
| 89 | INSR | 00975 | 0.1118 | 0.0090 |
| 90 | FKBP8 | 05327 | 0.1110 | 0.0093 |
| 91 | BAK1 | 02744 | 0.1078 | 0.0095 |
| 92 | PDE3B | 03626 | 0.1076 | 0.0102 |
| 93 | CASP3 | 02799 | 0.1036 | 0.0100 |
| 94 | BBC3 | 16165 | 0.1033 | 0.0093 |
| 95 | CBX4 | 04357 | 0.1028 | 0.0099 |
| 96 | BCL2L10 | 06071 | 0.1025 | 0.0091 |
| 97 | BCL2L2 | 03569 | 0.1020 | 0.0106 |
| 98 | NDC80 | 06277 | 0.1018 | 0.0099 |
| 99 | GFI1B | 05088 | 0.1016 | 0.0098 |
| 100 | INTS4 | 12386 | 0.1009 | 0.0103 |
| 101 | BMF | 05881 | 0.1004 | 0.0104 |
| 102 | AKTIP | 07467 | 0.0988 | 0.0103 |
| 103 | PKMYT1 | 03920 | 0.0949 | 0.0108 |
| 104 | MLL | 01162 | 0.0932 | 0.0097 |
| 105 | BAG4 | 04861 | 0.0931 | 0.0109 |
| 106 | BCL10 | 04625 | 0.0920 | 0.0110 |
| 107 | BMI1 | 01277 | 0.0912 | 0.0108 |
| 108 | PCGF2 | 10621 | 0.0897 | 0.0114 |
| 109 | TSC22D4 | 18231 | 0.0869 | 0.0102 |
| 110 | YWHAQ | 00886 | 0.0862 | 0.0113 |
| 111 | PRG2 | 09283 | 0.0860 | 0.0117 |

**Table 6.** The top 100 ranked proteins using VAVIEN with 11 seeds (flagged by *) in ADR sutdy

| **Rank** | **Gene Symbol** | **HPRD ID** | **Score** |
| --- | --- | --- | --- |
| 1 | ABCB1* | 01370 | 1.1525 |
| 2 | ABCB11* | 04436 | 1.1520 |
| 3 | BAD* | 04409 | 1.0745 |
| 4 | AKT1* | 01261 | 1.0709 |
| 5 | BCL2* | 01045 | 1.0500 |
| 6 | TERT* | 01754 | 1.0447 |
| 7 | TOP2A* | 00536 | 1.0125 |
| 8 | MAD2L1* | 03274 | 1.0040 |
| 9 | ZNRD1* | 09601 | 1.0016 |
| 10 | ABCB8* | 10400 | 0.9989 |
| 11 | CYP3A4* | 00484 | 0.9968 |
| 12 | UGT2B7 | 02508 | 0.8791 |
| 13 | HAX1 | 12075 | 0.8539 |
| 14 | PIM3 | 15137 | 0.5768 |
| 15 | DLC1 | 05035 | 0.5725 |
| 16 | CCDC88A | 11131 | 0.5725 |
| 17 | TCL6 | 05104 | 0.5725 |
| 18 | THEM4 | 16215 | 0.5725 |
| 19 | UXS1 | 15642 | 0.5725 |
| 20 | TP53AIP1 | 10397 | 0.5685 |
| 21 | CPT1A | 02755 | 0.5685 |
| 22 | ABCB4 | 01371 | 0.4976 |
| 23 | SMG6 | 06502 | 0.3716 |
| 24 | SPNS1 | 11599 | 0.3471 |
| 25 | BCL2L14 | 05841 | 0.3471 |
| 26 | TPP1 | 06415 | 0.3249 |
| 27 | ITM2B | 04878 | 0.3148 |
| 28 | HRK | 04581 | 0.2863 |
| 29 | SACS | 05135 | 0.2822 |
| 30 | C4orf14 | 12823 | 0.2768 |
| 31 | RNF2 | 07028 | 0.2671 |
| 32 | TMBIM6 | 02851 | 0.2483 |
| 33 | NMT2 | 11948 | 0.2418 |
| 34 | UBD | 09354 | 0.2415 |
| 35 | MTCP1 | 02122 | 0.2362 |
| 36 | PDE3B | 03626 | 0.2344 |
| 37 | TCL1B | 04795 | 0.2297 |
| 38 | SAT1 | 02431 | 0.2281 |
| 39 | METTL1 | 06834 | 0.2266 |
| 40 | BFAR | 16548 | 0.2249 |
| 41 | CAV1 | 03028 | 0.2243 |
| 42 | BCL2L10 | 06071 | 0.2236 |
| 43 | KIAA0513 | 17192 | 0.2214 |
| 44 | TEP1 | 03404 | 0.2123 |
| 45 | FKBP8 | 05327 | 0.2066 |
| 46 | BIK | 04547 | 0.2032 |
| 47 | SMG5 | 16869 | 0.2028 |
| 48 | CIC | 10831 | 0.1924 |
| 49 | NONO | 02098 | 0.1895 |
| 50 | DFFB | 03532 | 0.1848 |
| 51 | BCL2L2 | 03569 | 0.1845 |
| 52 | BBC3 | 16165 | 0.1819 |
| 53 | KRT10 | 01021 | 0.1745 |
| 54 | IMPDH2 | 00895 | 0.1736 |
| 55 | AHNAK | 14684 | 0.1659 |
| 56 | BMF | 05881 | 0.1657 |
| 57 | ATXN2 | 03307 | 0.1639 |
| 58 | - | 05934 | 0.1620 |
| 59 | BCLAF1 | 16544 | 0.1599 |
| 60 | WDR18 | 18296 | 0.1580 |
| 61 | BECN1 | 05087 | 0.1565 |
| 62 | GFI1B | 05088 | 0.1483 |
| 63 | TCL1A | 01744 | 0.1480 |
| 64 | BCL2A1 | 03034 | 0.1470 |
| 65 | DGKD | 03492 | 0.1464 |
| 66 | WNK1 | 05570 | 0.1459 |
| 67 | BCL2L11 | 04828 | 0.1449 |
| 68 | PMAIP1 | 12002 | 0.1433 |
| 69 | BAG4 | 04861 | 0.1412 |
| 70 | PPARGC1B | 10594 | 0.1394 |
| 71 | AKT1S1 | 12441 | 0.1394 |
| 72 | HMOX1 | 00782 | 0.1357 |
| 73 | BCL2L1 | 02497 | 0.1356 |
| 74 | ANAPC2 | 07367 | 0.1352 |
| 75 | PKMYT1 | 03920 | 0.1339 |
| 76 | BNIP1 | 04480 | 0.1320 |
| 77 | BNIPL | 16557 | 0.1283 |
| 78 | YWHAQ | 00886 | 0.1275 |
| 79 | INTS4 | 12386 | 0.1269 |
| 80 | BNIP3 | 04482 | 0.1262 |
| 81 | INO80C | 10719 | 0.1247 |
| 82 | TACO1 | 17421 | 0.1247 |
| 83 | RBM17 | 09501 | 0.1247 |
| 84 | TP53AP1 | 10276 | 0.1247 |
| 85 | FAM86C | 13337 | 0.1247 |
| 86 | WDR4 | 09330 | 0.1221 |
| 87 | MAD2L2 | 07246 | 0.1203 |
| 88 | RAB3D | 05070 | 0.1200 |
| 89 | HTR1F | 01637 | 0.1186 |
| 90 | S1PR1 | 03578 | 0.1177 |
| 91 | CDC16 | 04587 | 0.1159 |
| 92 | MTOR | 03134 | 0.1146 |
| 93 | TOP2B | 00537 | 0.1138 |
| 94 | CDC27 | 00304 | 0.1119 |
| 95 | MBL2 | 01107 | 0.1110 |
| 96 | RUVBL1 | 09143 | 0.1096 |
| 97 | BNIP3L | 07288 | 0.1080 |
| 98 | NMB | 01210 | 0.1071 |
| 99 | ATP2A2 | 00161 | 0.1063 |
| 100 | PFKFB2 | 01383 | 0.1059 |
| 101 | CAMKK1 | 13000 | 0.1054 |
| 102 | EIF3F | 04887 | 0.1052 |
| 103 | PAK7 | 06423 | 0.1051 |
| 104 | PRKCA | 01498 | 0.1041 |
| 105 | MAD2L1BP | 10065 | 0.1041 |
| 106 | PPP3CA | 00234 | 0.1039 |
| 107 | PRG2 | 09283 | 0.1038 |
| 108 | BNIP2 | 04481 | 0.1037 |
| 109 | ADAM17 | 04703 | 0.1036 |
| 110 | BAK1 | 02744 | 0.1017 |
| 111 | PIM2 | 02244 | 0.1015 |

**Table 7.** ProteinRank scores for the 12 seed proteins (flagged by *) and the top 100 ranked proteins in VCR study

| **Rank** | **Gene Symbol** | **HPRD ID** | **RankScore** | **P Value** |
| --- | --- | --- | --- | --- |
| 1 | ABCB1* | 01370 | 1.1752 | 0.0000 |
| 2 | ABCB11* | 04436 | 1.1576 | 0.0001 |
| 3 | ABCC2* | 03065 | 1.0921 | 0.0002 |
| 4 | SLC22A3* | 05328 | 1.0886 | 0.0002 |
| 5 | TUBB* | 01852 | 1.0850 | 0.0006 |
| 6 | TUBA4A* | 01851 | 1.0846 | 0.0006 |
| 7 | SRI* | 01680 | 1.0360 | 0.0006 |
| 8 | AKT1* | 01261 | 1.0256 | 0.0007 |
| 9 | ZNRD1* | 09601 | 1.0091 | 0.0009 |
| 10 | CYP3A4* | 00484 | 1.0074 | 0.0009 |
| 11 | PPP1R1B* | 05097 | 1.0043 | 0.0011 |
| 12 | RALBP1* | 09013 | 1.0035 | 0.0009 |
| 13 | HAX1 | 12075 | 0.9528 | 0.0015 |
| 14 | RNF2 | 07028 | 0.7201 | 0.0014 |
| 15 | PDZD3 | 06191 | 0.6316 | 0.0015 |
| 16 | TTK | 04973 | 0.5275 | 0.0016 |
| 17 | CAV1 | 03028 | 0.4768 | 0.0017 |
| 18 | ANXA11 | 03983 | 0.4337 | 0.0018 |
| 19 | RYR2 | 01619 | 0.4054 | 0.0015 |
| 20 | CALCOCO2 | 06846 | 0.3975 | 0.0019 |
| 21 | CACNA1C | 00246 | 0.3706 | 0.0023 |
| 22 | SHBG | 01646 | 0.3656 | 0.0015 |
| 23 | B4GALT1 | 00659 | 0.3577 | 0.0024 |
| 24 | C5orf25 | 13486 | 0.3551 | 0.0025 |
| 25 | CACNA1S | 00248 | 0.3509 | 0.0025 |
| 26 | TBCE | 05381 | 0.3418 | 0.0028 |
| 27 | LGALS2 | 08859 | 0.3392 | 0.0024 |
| 28 | SMAD2 | 03221 | 0.3314 | 0.0027 |
| 29 | CALB2 | 00230 | 0.3243 | 0.0029 |
| 30 | GCA | 06122 | 0.3217 | 0.0034 |
| 31 | CANX | 00252 | 0.3194 | 0.0034 |
| 32 | TAOK2 | 18147 | 0.3084 | 0.0030 |
| 33 | NONO | 02098 | 0.2910 | 0.0033 |
| 34 | CFTR | 03883 | 0.2747 | 0.0033 |
| 35 | MAP6 | 15989 | 0.2628 | 0.0034 |
| 36 | TBCB | 03196 | 0.2549 | 0.0034 |
| 37 | ANXA7 | 01720 | 0.2461 | 0.0038 |
| 38 | TUBB1 | 18243 | 0.2439 | 0.0033 |
| 39 | AZU1 | 01221 | 0.2439 | 0.0038 |
| 40 | PSEN2 | 02860 | 0.2438 | 0.0039 |
| 41 | METTL1 | 06834 | 0.2428 | 0.0037 |
| 42 | PLXNA1 | 11868 | 0.2347 | 0.0043 |
| 43 | PCGF6 | 07427 | 0.2322 | 0.0040 |
| 44 | HMOX1 | 00782 | 0.2305 | 0.0046 |
| 45 | TTBK1 | 11653 | 0.2289 | 0.0047 |
| 46 | TCL6 | 05104 | 0.2208 | 0.0047 |
| 47 | UXS1 | 15642 | 0.2208 | 0.0050 |
| 48 | DLC1 | 05035 | 0.2208 | 0.0049 |
| 49 | CCDC88A | 11131 | 0.2208 | 0.0053 |
| 50 | THEM4 | 16215 | 0.2208 | 0.0044 |
| 51 | IMPDH2 | 00895 | 0.2162 | 0.0049 |
| 52 | AHNAK | 14684 | 0.2121 | 0.0046 |
| 53 | TCL1B | 04795 | 0.2073 | 0.0051 |
| 54 | MYST1 | 11381 | 0.1986 | 0.0064 |
| 55 | YWHAG | 05639 | 0.1950 | 0.0065 |
| 56 | SIRT2 | 10377 | 0.1859 | 0.0057 |
| 57 | KIAA0513 | 17192 | 0.1844 | 0.0064 |
| 58 | ATXN2 | 03307 | 0.1809 | 0.0061 |
| 59 | TCL1A | 01744 | 0.1797 | 0.0066 |
| 60 | PDK2 | 03955 | 0.1765 | 0.0061 |
| 61 | LTA | 01084 | 0.1735 | 0.0063 |
| 62 | RING1 | 03624 | 0.1720 | 0.0064 |
| 63 | RTN4 | 07259 | 0.1690 | 0.0065 |
| 64 | RAB3D | 05070 | 0.1669 | 0.0067 |
| 65 | VEGFA | 01889 | 0.1641 | 0.0065 |
| 66 | ABCB4 | 01371 | 0.1630 | 0.0060 |
| 67 | BMPR2 | 02880 | 0.1613 | 0.0072 |
| 68 | RYBP | 09607 | 0.1571 | 0.0068 |
| 69 | FKBP1B | 02795 | 0.1557 | 0.0063 |
| 70 | TOPBP1 | 09678 | 0.1556 | 0.0070 |
| 71 | CSNK2A1 | 00277 | 0.1545 | 0.0076 |
| 72 | TFCP2 | 01790 | 0.1542 | 0.0071 |
| 73 | ARL8B | 12483 | 0.1524 | 0.0081 |
| 74 | SVIL | 04992 | 0.1468 | 0.0069 |
| 75 | PRKDC | 02941 | 0.1424 | 0.0082 |
| 76 | MTCP1 | 02122 | 0.1409 | 0.0074 |
| 77 | PKD2 | 01437 | 0.1333 | 0.0079 |
| 78 | PRG2 | 09283 | 0.1327 | 0.0081 |
| 79 | APPL1 | 05053 | 0.1310 | 0.0079 |
| 80 | AKAP6 | 05257 | 0.1306 | 0.0084 |
| 81 | KRT10 | 01021 | 0.1300 | 0.0075 |
| 82 | CACNB3 | 11879 | 0.1285 | 0.0084 |
| 83 | PPARGC1B | 10594 | 0.1278 | 0.0086 |
| 84 | PRKCA | 01498 | 0.1276 | 0.0079 |
| 85 | WDR4 | 09330 | 0.1258 | 0.0082 |
| 86 | IL1A | 00988 | 0.1252 | 0.0081 |
| 87 | CIC | 10831 | 0.1249 | 0.0084 |
| 88 | PDCD6 | 03035 | 0.1245 | 0.0094 |
| 89 | RAB8B | 11476 | 0.1230 | 0.0084 |
| 90 | SEMA6D | 10221 | 0.1213 | 0.0088 |
| 91 | DYNLL1 | 03334 | 0.1210 | 0.0097 |
| 92 | BLVRB | 02967 | 0.1192 | 0.0089 |
| 93 | ALG2 | 09716 | 0.1189 | 0.0090 |
| 94 | PHC1 | 16009 | 0.1184 | 0.0092 |
| 95 | TBCD | 05227 | 0.1183 | 0.0097 |
| 96 | BCL10 | 04625 | 0.1181 | 0.0103 |
| 97 | KDM5D | 02464 | 0.1174 | 0.0094 |
| 98 | STARD13 | 11607 | 0.1167 | 0.0101 |
| 99 | S1PR1 | 03578 | 0.1166 | 0.0104 |
| 100 | SYT9 | 11618 | 0.1164 | 0.0113 |
| 101 | ALDOA | 00070 | 0.1164 | 0.0093 |
| 102 | NOS3 | 01224 | 0.1157 | 0.0105 |
| 103 | CBX4 | 04357 | 0.1116 | 0.0100 |
| 104 | FLNA | 02060 | 0.1113 | 0.0107 |
| 105 | CSNK2A2 | 00279 | 0.1112 | 0.0105 |
| 106 | EZH2 | 03342 | 0.1110 | 0.0099 |
| 107 | CPNE7 | 05749 | 0.1106 | 0.0108 |
| 108 | PCGF1 | 11404 | 0.1106 | 0.0103 |
| 109 | DBNDD2 | 12763 | 0.1106 | 0.0107 |
| 110 | YWHAZ | 03183 | 0.1102 | 0.0111 |
| 111 | ILK | 03842 | 0.1062 | 0.0114 |
| 112 | RAC1 | 03627 | 0.1051 | 0.0110 |

**Table 8.** The top 100 ranked proteins using VAVIEN with 12 seeds (flagged by *) in VCR sutdy

| **Rank** | **Gene Symbol** | **HPRD ID** | **Score** |
| --- | --- | --- | --- |
| 1 | ABCB1* | 01370 | 1.1520 |
| 2 | ABCB11* | 04436 | 1.1495 |
| 3 | ABCC2* | 03065 | 1.1281 |
| 4 | SLC22A3* | 05328 | 1.1264 |
| 5 | TUBA4A* | 01851 | 1.0305 |
| 6 | TUBB* | 01852 | 1.0298 |
| 7 | AKT1* | 01261 | 1.0099 |
| 8 | RALBP1* | 09013 | 1.0048 |
| 9 | PPP1R1B* | 05097 | 1.0042 |
| 10 | SRI* | 01680 | 1.0026 |
| 11 | ZNRD1* | 09601 | 0.9986 |
| 12 | CYP3A4* | 00484 | 0.9964 |
| 13 | UGT2B7 | 02508 | 0.8788 |
| 14 | HAX1 | 12075 | 0.8497 |
| 15 | PDZD3 | 06191 | 0.8301 |
| 16 | C5orf25 | 13486 | 0.5568 |
| 17 | DLC1 | 05035 | 0.5396 |
| 18 | CCDC88A | 11131 | 0.5396 |
| 19 | TCL6 | 05104 | 0.5396 |
| 20 | THEM4 | 16215 | 0.5396 |
| 21 | UXS1 | 15642 | 0.5396 |
| 22 | SLC15A1 | 02768 | 0.5075 |
| 23 | SLCO1C1 | 08342 | 0.5075 |
| 24 | ABCB4 | 01371 | 0.4951 |
| 25 | SLC15A2 | 09088 | 0.3704 |
| 26 | TBCE | 05381 | 0.3431 |
| 27 | PFDN4 | 05358 | 0.3364 |
| 28 | CALB2 | 00230 | 0.3086 |
| 29 | B4GALT1 | 00659 | 0.2805 |
| 30 | SLCO3A1 | 07502 | 0.2788 |
| 31 | SLC22A9 | 06982 | 0.2788 |
| 32 | SACS | 05135 | 0.2709 |
| 33 | CACNA1S | 00248 | 0.2667 |
| 34 | RNF2 | 07028 | 0.2664 |
| 35 | SLC22A5 | 04539 | 0.2641 |
| 36 | SLCO1A2 | 09112 | 0.2641 |
| 37 | SLC22A4 | 09171 | 0.2641 |
| 38 | RAB8B | 11476 | 0.2476 |
| 39 | PDZK1 | 04829 | 0.2444 |
| 40 | LGALS2 | 08859 | 0.2395 |
| 41 | NCALD | 09466 | 0.2372 |
| 42 | TAOK2 | 18147 | 0.2352 |
| 43 | PDE3B | 03626 | 0.2344 |
| 44 | GUCY2C | 07529 | 0.2323 |
| 45 | CAV1 | 03028 | 0.2278 |
| 46 | SLC9A3R1 | 05406 | 0.2198 |
| 47 | KIAA0513 | 17192 | 0.2188 |
| 48 | TTK | 04973 | 0.2174 |
| 49 | TBCB | 03196 | 0.2172 |
| 50 | MTCP1 | 02122 | 0.2165 |
| 51 | TCL1B | 04795 | 0.2158 |
| 52 | METTL1 | 06834 | 0.2048 |
| 53 | GCA | 06122 | 0.2009 |
| 54 | RDX | 01534 | 0.1932 |
| 55 | CIC | 10831 | 0.1926 |
| 56 | RALB | 01550 | 0.1790 |
| 57 | NONO | 02098 | 0.1789 |
| 58 | ANXA7 | 01720 | 0.1732 |
| 59 | TTBK1 | 11653 | 0.1702 |
| 60 | C4orf17 | 12825 | 0.1691 |
| 61 | ATXN2 | 03307 | 0.1674 |
| 62 | ARL8B | 12483 | 0.1671 |
| 63 | ROBO2 | 11895 | 0.1643 |
| 64 | CACNA1C | 00246 | 0.1639 |
| 65 | KRT10 | 01021 | 0.1625 |
| 66 | IMPDH2 | 00895 | 0.1597 |
| 67 | REPS1 | 09687 | 0.1572 |
| 68 | AHNAK | 14684 | 0.1565 |
| 69 | ANXA11 | 03983 | 0.1551 |
| 70 | TBCD | 05227 | 0.1548 |
| 71 | MAP1LC3A | 03144 | 0.1499 |
| 72 | CANX | 00252 | 0.1473 |
| 73 | TCL1A | 01744 | 0.1467 |
| 74 | GFI1B | 05088 | 0.1448 |
| 75 | DGKD | 03492 | 0.1424 |
| 76 | DLGAP4 | 16826 | 0.1423 |
| 77 | SLC22A12 | 06160 | 0.1394 |
| 78 | SLC17A1 | 01661 | 0.1394 |
| 79 | SLC26A6 | 10235 | 0.1394 |
| 80 | WNK1 | 05570 | 0.1359 |
| 81 | RYR2 | 01619 | 0.1348 |
| 82 | SYNJ2BP | 15455 | 0.1328 |
| 83 | PPARGC1B | 10594 | 0.1316 |
| 84 | GUCA2A | 00767 | 0.1311 |
| 85 | TUBB1 | 18243 | 0.1292 |
| 86 | AZU1 | 01221 | 0.1292 |
| 87 | HMOX1 | 00782 | 0.1273 |
| 88 | REPS2 | 02262 | 0.1264 |
| 89 | AKT1S1 | 12441 | 0.1255 |
| 90 | INTS4 | 12386 | 0.1252 |
| 91 | PRKACA | 03382 | 0.1234 |
| 92 | HTR1F | 01637 | 0.1203 |
| 93 | RAB3D | 05070 | 0.1197 |
| 94 | SLC26A3 | 00544 | 0.1189 |
| 95 | TCP1 | 01748 | 0.1188 |
| 96 | MAP6 | 15989 | 0.1184 |
| 97 | MARK4 | 09402 | 0.1174 |
| 98 | TM4SF1 | 01854 | 0.1173 |
| 99 | HDAC6 | 02228 | 0.1148 |
| 100 | SIRT2 | 10377 | 0.1142 |
| 101 | CSNK2A1 | 00277 | 0.1134 |
| 102 | S1PR1 | 03578 | 0.1129 |
| 103 | CSNK2A2 | 00279 | 0.1118 |
| 104 | WDR4 | 09330 | 0.1099 |
| 105 | DPYSL2 | 03914 | 0.1093 |
| 106 | CDK5 | 00449 | 0.1087 |
| 107 | PPP1CA | 15942 | 0.1087 |
| 108 | LTA | 01084 | 0.1075 |
| 109 | CRIPT | 05208 | 0.1073 |
| 110 | SYT9 | 11618 | 0.1053 |
| 111 | MAP4 | 01141 | 0.1048 |
| 112 | EIF3F | 04887 | 0.1046 |

**Supplementary Figures：**


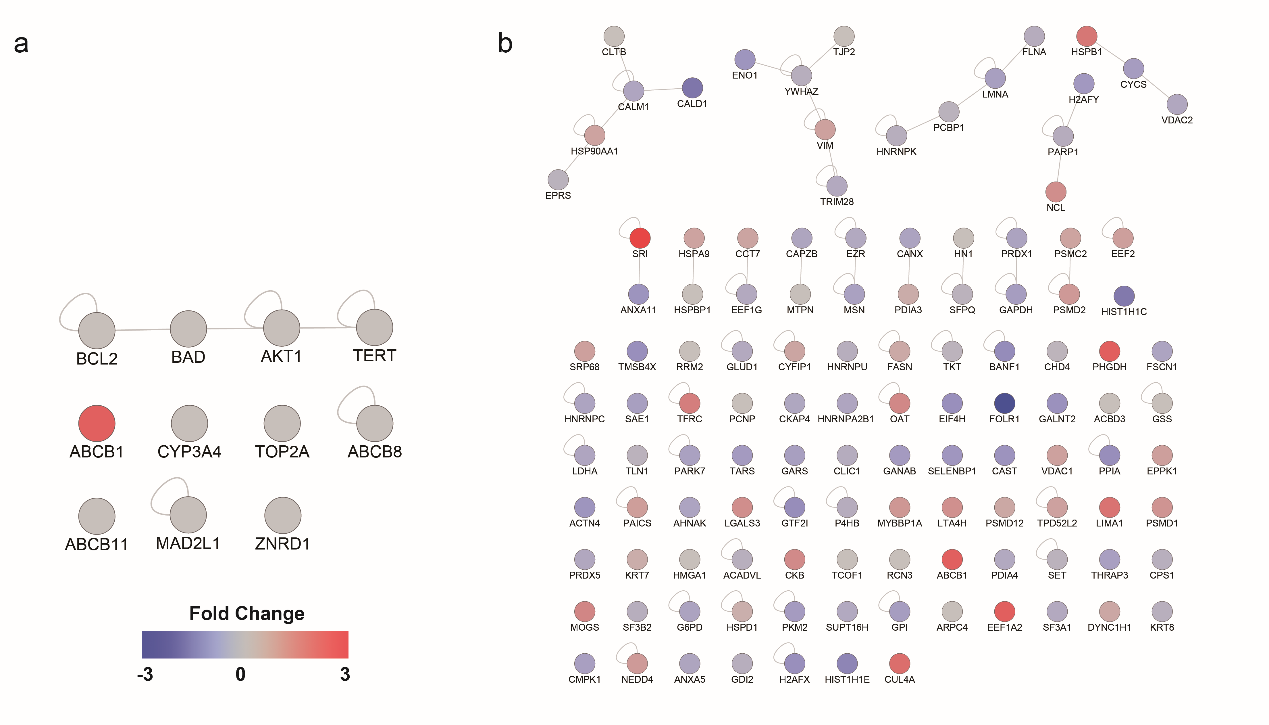


**Figure 1.** Expression levels and interactions among 11 ADR seeds (A) and 119 differentially expressed proteins in ADR resistant GCCs (B). Out of 130 differentially expressed proteins identified by MS analysis, 119 were included in this PPI network. Fold changes were log2 transformed.


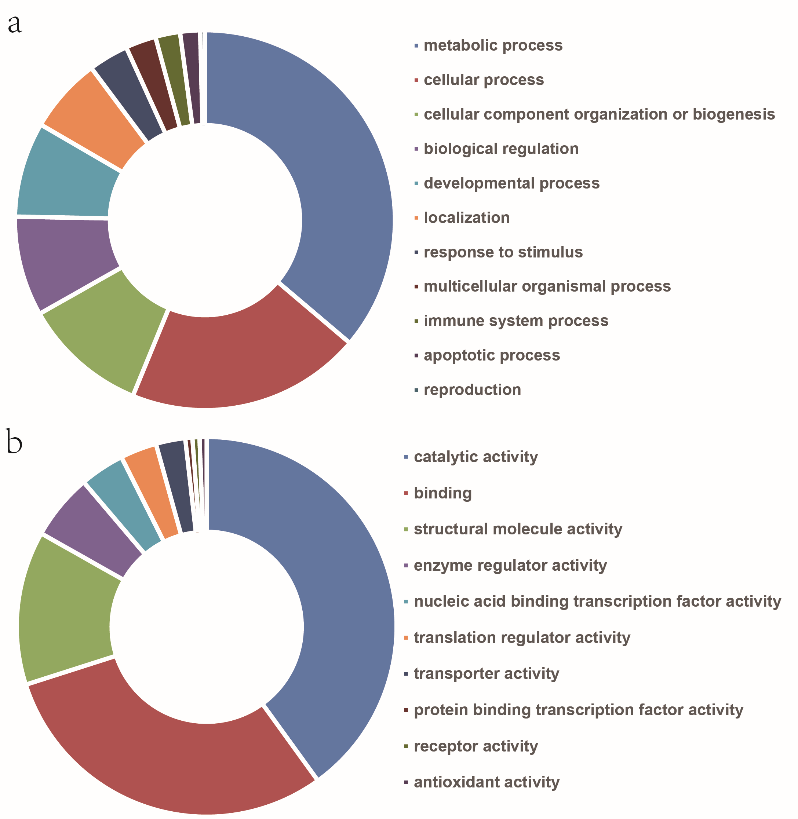


**Figure 2.** An analysis of the 130 differentially expressed proteins in SGC7901/ADR compared to SGC7901 identified by mass spectrometry using the PANTHER classification system. The categorization was based on the (a) Biological process and (b) Molecular function provided by PANTHER
